# Amyotrophic Lateral Sclerosis associated FUS mutation shortens mitochondria and induces neurotoxicity

**DOI:** 10.1038/s41598-018-33964-0

**Published:** 2018-10-22

**Authors:** Tadashi Nakaya, Manolis Maragkakis

**Affiliations:** 1 Laboratory of Neuroscience, Graduate School of Pharmaceutical Sciences, Hokkaido University, Sapporo, 060-0812 Japan; 2 Department of Pathology and Laboratory Medicine, Division of Neuropathology, University of Pennsylvania, Philadelphia, Pennsylvania 19104 USA; 3 Institute for Translational Medicine and Therapeutics, University of Pennsylvania, Philadelphia, Pennsylvania 19104 USA; 4 Penn Medicine Translational Neuroscience Center, Perelman School of Medicine, University of Pennsylvania, Philadelphia, Pennsylvania 19104 USA

## Abstract

Amyotrophic Lateral Sclerosis (ALS) is a lethal neurodegenerative disorder that primarily affects motor neurons. Dominant mutations in the RNA binding protein Fused in Sarcoma (FUS) have been identified as causative factors of ALS. Mutation, R495X, results in a premature stop codon and induces an aggressive disease phenotype by a largely unknown process. Here, we employ CLIP-Seq, RNA-Seq and Ribo-Seq in cultured neurons expressing R495X or wild-type FUS to identify the mutation effects on the FUS targetome and on the neuronal transcriptome at the expression and translation level, simultaneously. We report that, unlike wild-type FUS that binds on precursor mRNAs (pre-mRNAs), R495X binds mature mRNAs in the cytoplasm. R495X has a moderate effect on target mRNA expression and its binding induces only modest expression changes. In contrast, we find that R495X controls the translation of genes that are associated with mitochondria function and results in significant reduction of mitochondrial size. Importantly, we show that introduction of the 4FL mutation that alters binding of R495X to RNA, partially abrogates R495X-induced effects on mRNA translation, mitochondrial size and neurotoxicity. Our findings uncover a novel RNA-mediated pathway of FUS R495X-induced neurotoxicity that affects mitochondria morphology and provide insight to previous studies associating mitochondria dysfunction to ALS.

## Introduction

Amyotrophic Lateral Sclerosis (ALS) is a lethal neurological disease characterized by progressive neurodegeneration of the upper and lower motor neurons^1^. Patients usually progress to fatal paralysis and die from dysfunction of the respiratory system. Dominant mutations in *Fused in Sarcoma* (*FUS*) gene have been identified in familial cases of ALS (FALS)^2–4^. *FUS* encodes an RNA binding protein composed of 526 amino acids; it localizes primarily to the nucleus and consists of seven domains: QGSY-rich, Gly-rich, RNA recognition motif (RRM), Arg-Gly-Gly1 (RGG1), Zinc finger (ZnF), RGG2 and nuclear localization signal (NLS)^4^. The majority of the disease-causing mutations localize at the C-terminal region, disrupt the NLS and result in abnormal FUS cytoplasmic localization. FUS mislocalization has been hypothesized to disrupt its nuclear function and promote gain of function in the cytoplasm of affected neurons^5–7^. R495X, a FUS mutation in which the Arginine at position 495 changes into a premature stop codon, results in complete loss of the NLS and shows a severe disease phenotype^8,9^. Despite the severity of the phenotype, the mechanistic effects and the implications in neurons are still unknown.

Previously, we uncovered the genome-wide RNA binding profile of FUS in neurons and reported that it preferentially binds evolutionarily conserved introns of other RNA-binding proteins, altering their expression^10^. Similarly, other groups have shown that FUS controls the mRNA splicing of its target genes^6,11–15^. However, these studies identified only minor phenotypic effects, even after FUS knockdown/knockout experiments, raising the possibility that the disease phenotype is the result of a gain instead of loss of function. Overexpression of wild-type FUS in mice, *Drosophila melanogaster* and rats results in neurotoxicity^16–18^, while disease mutant FUS causes motor neuron degeneration via toxic gain of function in mice^19^. Furthermore, the gene expression profile that results from overexpression of the disease-associated FUS mutant in HEK293T cells is closer to that of wild-type FUS overexpression than knockdown^20^ indicating that cytoplasmic accumulation results in a gain of function. This is further supported by the inability of wild-type FUS to rescue mutant FUS phenotype in *C*. *elegans*^21^.

Recently, several reports demonstrated that FUS can form liquid droplets that are separated from solution *in vitro* and *in vivo*. The formation of these droplets is a reversible process that is dependent on the FUS low complexity domain^22,23^, concentration and incubation temperature. FUS mutations have been shown to accelerate the liquid-to-solid phase transition converting reversible to irreversible hydrogels^24,25^ and impairing ribonucleoprotein granule formation. However, it is still unclear how this relates to the disease phenotype and how FUS gain of function leads to neurodegeneration.

To examine the aggressive disease phenotype of FUS R495X and identify the involved molecular pathways, we probed its target RNA repertoire and the gene expression and translation in neurons. We find that R495X has an altered gene binding profile which leads to neurotoxicity, perturbs translation of transcripts associated with mitochondrial function and causes reduction of the mitochondria size. By altering the binding between RNA targets and R495X, we reveal a partial abrogation of the effects of the mutation, supporting RNA dysregulation as an important player in FUS R495X mediated neurotoxicity.

## Results

### FUS R495X mutant mislocalizes to the cytoplasm

**Figure 1:**
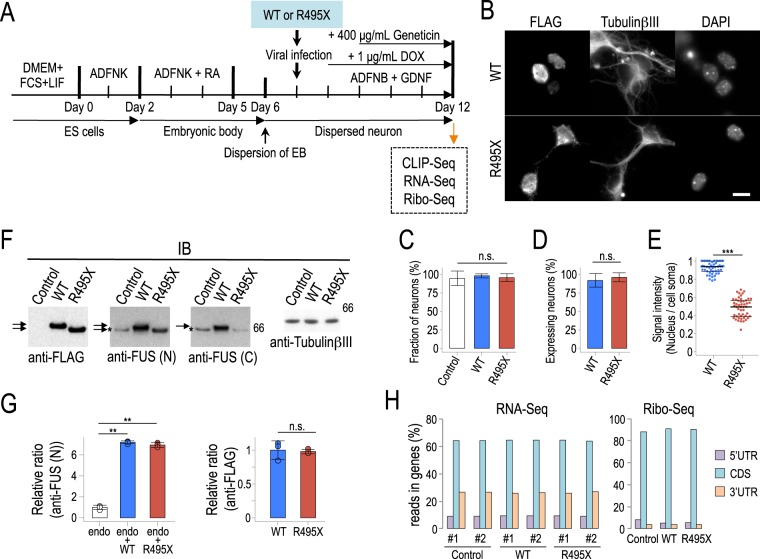
CLIP-Seq, RNA-Seq and Ribo-Seq on human FUS WT- or R495X-expressing neurons. (**A**) Schematic of the experimental design for CLIP-Seq, RNA-Seq and Ribo-Seq library preparation from mouse ES cell derived neurons. (**B**) Immunostaining for cellular localization of NFLAG-hFUSWT (WT) and NFLAG-hFUSR495X (R495X) in neurons. Anti-FLAG (left), anti-TubulinβIII (middle) and DAPI stain (right) are shown. Scale bar, 10 μm. (**C**) Bar plot for fraction of neurons over all cells for Control, WT- and R495X-expressing cells. Error bars indicate standard deviation (N = 18). n.s., not significant (*p* = 0.253), one-way ANOVA test. (**D**) Bar plot for percentage of neurons expressing WT and R495X. Error bars indicate standard deviation (N = 18). n.s., not significant (*p* = 0.115), two-tailed Student’s *t*-test. (**E**). Beeswarm plot for ratio of nuclear localization of WT and R495X in neurons. Ratio represents fluorescence intensity in the nucleus over cell soma. N = 50. ^***^p = 2.83 × 10^−40^, two-tailed Student’s *t*-test. Black bars represent median, 25th and 75th percentiles. (**F**) Immunoblots showing expression levels using the indicated antibodies for FLAG, amino- (FUS(N)) and carboxyl-terminal FUS (FUS(C)) and TubulinβIII. Arrows and asterisk indicate exogenous and endogenous FUS proteins, respectively. Protein standard is shown on the right. (**G**) Quantification of endogenous (endo) and exogenous WT and R495X FUS proteins detected by anti-FUS (N) (left) and anti-FLAG (right). N = 3. ^**^p < 0.0001 one-way ANOVA and *p* < 0.01 in post Tukey’s HSD test for left graph. n.s., not significant (*p* = 0.827) two-tailed Student’s *t*-test for right graph. (**H**) Distribution of gene mapping reads in the 5′UTR, coding sequence (CDS) and 3′UTR for RNA-Seq and Ribo-Seq.

To investigate the consequences of the R495X mutant in neurons, we set up an inducible expression system based on lentiviral transduction of FLAG-tagged human R495X (NFLAG-hFUSR495X, R495X) or wild-type FUS (NFLAG-hFUSWT, WT) in neurons that we differentiated from mouse Embryonic Stem (ES) cells^10^. After doxycycline (DOX) induction and Geneticin selection we obtain a highly enriched population of neurons expressing the transgenes (Fig. 1A). We analyzed the localization of exogenous proteins by immunostaining using anti-FLAG along with anti-TubulinβIII antibody and DAPI staining (Fig. 1B). We confirmed that the fraction of neurons was unaffected (Fig. 1C, Supplementary Fig. S1) by WT or R495X overexpression and that the exogenous proteins were expressed in the majority of neurons (Fig. 1D, Supplementary Fig. S1) highlighting the quality of our selection process. We found that while WT localized to the nucleus, R495X mislocalized to the cytoplasm (Fig. 1B), as expected due to loss of NLS, and consistent with a previous report^9^. Quantification of the fluorescence intensity showed that 93% of WT and only 48% of R495X was detected in the nuclei (Fig. 1E).

### Probing the FUS targetome, gene expression and translation in R495X-expressing neurons

To evaluate R495X effects on the RNA target repertoire and on gene expression and translation levels, we performed high-throughput sequencing after crosslinking and immunoprecipitation (CLIP-Seq), RNA-Seq and Ribosome profiling (Ribo-Seq) in neurons expressing WT or R495X. First, we analyzed the expression levels of the exogenous proteins by immunoblot using anti-FLAG, anti-FUS amino-terminal, and anti-FUS carboxyl-terminal antibodies (Fig. 1F). Anti-FLAG antibody showed that R495X migrated faster than WT, consistent with its shorter length. The anti-FUS amino-terminal antibody recognized all proteins, including endogenous FUS, while the anti-FUS carboxyl-terminal antibody recognized WT and endogenous FUS but not R495X. Our results indicate that WT and R495X were expressed at similar levels, ~7 times higher than endogenous FUS (Fig. 1G). Consistent with a previous report^12^, we observed a reduction of endogenous mouse FUS protein in both WT and R495X expressing neurons (Fig. 1F and Supplementary Fig. S6), indicating that FUS autoregulation is active in our system. After CLIP of FLAG-tagged proteins and 5′end RNA radiolabeling, we observed a specific smear in both WT and R495X autoradiographs corresponding to bound and crosslinked RNA (Supplementary Fig. S2). More than 75% of CLIP sequenced reads mapped to the mouse genome for both WT and R495X, demonstrating the high CLIP specificity and showing that both exogenous proteins bound to RNA in neurons. Comparing FUS WT targetome to a control FUS CLIP library expressed at endogenous levels^10^ we found significant correlation (*R* = 0.6, Pearson’s correlation, p < 10^-16^) indicating that overexpression of the human FUS homolog only modestly affected gene binding. Similarly, for RNA-Seq, approximately 60% and 35% of the sequenced reads mapped in the coding sequence and UTRs, respectively, while the corresponding percentages for Ribo-Seq were 90% and 6% (Fig. 1H). This is consistent with the expectation of higher ribosome occupancy in the coding region and illustrates the protocol specificity.

### R495X binds mature mRNAs in contrast to WT that binds precursor mRNAs

**Figure 2:**
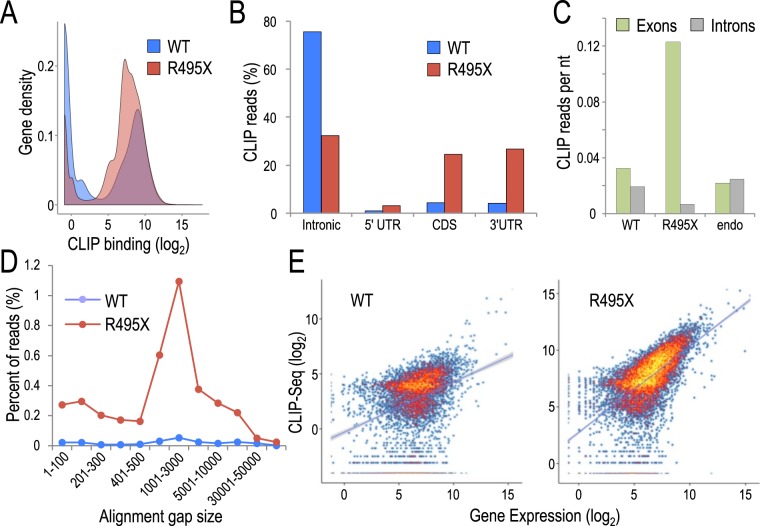
Identification of RNA binding property of R495X compared to WT. (**A**) Gene density plot of CLIP binding for WT (blue) and R495X (red). (**B**) Distribution of CLIP-Seq reads in introns, 5′UTR, coding sequence (CDS) and 3′UTR for WT (blue) and R495X (red). (**C**) Distribution of CLIP-Seq reads in exons (green) and introns (grey) normalized by the total length of these elements for WT, R495X and endogenous FUS (endo). (**D**). Alignment gap size distribution of CLIP-Seq reads for WT (blue) and R495X (red) reads. Only alignment gaps overlapping annotated introns are used. (**E**) Scatter plot for FUS binding versus gene expression levels for WT (left) and R495X (right). Color indicates gene density with yellow and blue indicating higher and lower values respectively.

Next we wished to identify potential differences in the gene targeting profile of WT and R495X. We found that R495X bound to substantially more genes than WT, likely due to its cytoplasmic localization (Fig. 2A). Analysis of the genic distribution of CLIP reads showed that more than ~75% of WT reads derived from introns, whereas only ~10% from exons (Fig. 2B). In contrast, only ~32% of R495X CLIP reads mapped in introns and ~54% in exons. To evaluate the binding preference for these regions, excluding stochastic differences attributed to their length, we normalized the number of reads in exons and introns by the aggregate length of these elements. We found that R495X bound substantially more on exonic regions, in contrast to WT and endogenous FUS^10^ that showed no binding preference (Fig. 2C).

Our findings indicate that, contrary to WT that binds on pre-mRNAs, R495X mostly binds on mature mRNAs and this is attributable to R495X mutation and not FUS overexpression. To further test this, we reasoned that R495X binding on mature mRNAs would be reflected by increased binding on exon-exon junctions. We quantified exon-exon junction binding by measuring the sequencing reads that align on the genome with alignment gap sizes of 1,000 to 3,000 nt, corresponding to spliced intronic sequences^26^. Comparing the two conditions, we found that R495X bound on exon-exon junctions at substantially higher levels than WT (Fig. 2D) further supporting that R495X mostly binds on mature mRNAs.

We hypothesized that R495X binding is mostly directed by protein localization in the cytoplasm and less by specific acquired binding preferences. We compared CLIP binding with gene expression levels and observed a substantially higher positive correlation (*R* = 0.61, Pearson’s correlation) for R495X than WT (*R* = 0.34, Pearson’s correlation) (Fig. 2E) indicating that R495X mostly binds on mRNAs in an expression-directed way. We also interrogated the CLIP data for overrepresented sequences using a 6mer enrichment approach^27^. Similar to previous reports^6,10–12,15^ we found modestly overrepresented sequences that only explained a small fraction of the CLIP reads (Supplementary Tables S1 and S2). Collectively, our results show a distinct RNA binding profile for the mutant, binding on mature mRNAs in the cytoplasm, guided mostly by protein localization and less by sequence-specificity.

### WT and R495X have modest effect on gene expression

**Figure 3:**
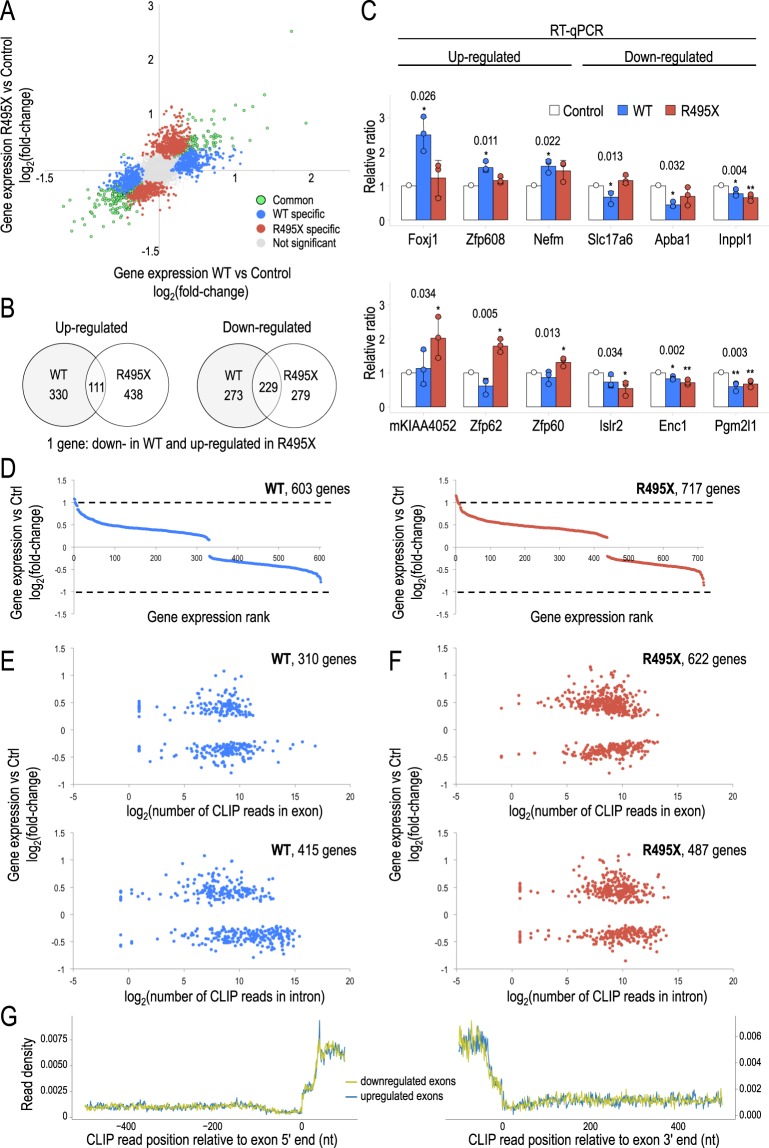
Differential gene expression analysis for WT- and R495X-expressing neurons. (**A**) Scatter plot of WT versus control and R495X versus control differential gene expression. Differential gene expression is quantified as log_2_(fold-change). (**B**) Venn diagram showing the number of up- (left) and down- (right) regulated genes in WT- and R495X-expressing neurons. (**C**) Bar plots of expression measured by RT-qPCR for up- (left) and down- (right) regulated genes that are differentially expressed in WT- (top) or R495X-expressing neurons (bottom). Values indicate P-values of one-way ANOVA test. ^*^p < 0.05, ^**^p < 0.01, post Tukey’s HSD test. Error bar indicates standard deviation (N = 3). (**D**) Gene differential expression for ranked list of differentially expressed genes for WT- (left) and R495X-expressing neurons (right). Dashed lines indicate 2-fold difference. (**E**,**F**) Scatter plot of gene differential expression versus FUS binding levels for WT (**E**) and R495X (**F**). FUS binding is quantified in exons (top) and introns (bottom). (**G**) R495X CLIP binding density upstream (left) and downstream (right) of up- and down-regulated exons. Position 0 corresponds to the 5′ end (left) and 3′ end (right) of exons.

To identify gene expression changes specifically induced by R495X and to deconvolute the mutation from the overexpression effect, we first probed gene expression changes in WT and R495X compared to a control library, prepared from non-transduced neurons, and focused on unique changes for each condition (Fig. 3A). Overall, at a significance level of 0.05, we identified 603 (up: 330 in Supplementary Table S3, down: 273 in Supplementary Table S4) and 717 (up: 438 in Supplementary Table S5, down: 279 in Supplementary Table S6) differentially expressed genes for WT and R495X compared to Control, respectively (Fig. 3B). We identified 340 (up: 111, down: 229) genes differentially expressed consistently in both conditions and only 1 gene in non-congruent direction between WT and R495X (Supplementary Table S7). We confirmed the observed changes by RT-qPCR for three, randomly selected, up- and down-regulated genes specific to WT or R495X (Fig. 3C). All tested genes showed significant expression changes in concordance with the RNA-Seq results. To gain insight into the potential function of the differentially expressed genes we performed gene ontology (GO) enrichment analysis for up- and down-regulated genes in WT (Supplementary Table S8) and R495X (Supplementary Table S9). WT was enriched for neuronal function terms such as ionotropic glutamate receptor signaling pathway whereas R495X for cellular processes such as regulation of cellular component organization.

Interestingly, we found that only 2 and 7 differentially expressed genes in WT and R495X, respectively, had expression changes higher than 2-fold compared to control. In both conditions, more than 89% (WT: 536/603, R495X: 670/717) of differentially expressed genes had differences smaller than 1.5-fold. To examine the effect of R495X on gene alternative splicing we analyzed the differential expression of exons and found only 110 exons on 99 genes with significant changes specific to R495X. Interestingly, the majority of them (81%) had less than 2-fold difference (Supplementary Table S10). Similarly, for WT we identified only 129 differentially expressed exons for 80 genes, 62% of which with less than 2-fold change (Supplementary Table S11). GO analysis for genes with differentially expressed exons in R495X-expressing neurons (Supplementary Table S12) revealed few enriched categories including second-messenger-mediated signaling, while more terms including cell-cell adhesion were enriched in WT-expressing neurons (Supplementary Table S13). Our results indicate that expression and splicing effects attributable either to overexpression or mutation are modest (Fig. 3D).

To further support this finding we tested whether the observed changes could be explained by R495X binding. To account for binding differences in exonic and intronic regions we quantified binding levels independently for the two classes. For WT, 310 and 415 of the 603 differentially expressed genes were bound in exons and introns, respectively (Fig. 3E, Supplementary Table S14). For R495X, the corresponding numbers were 622 and 487 of the 717 differentially expressed genes (Fig. 3F, Supplementary Table S15). In both conditions, comparing the differential expression with CLIP binding levels, we found no correlation (*R*^2^ < 0.2, Pearson’s correlation). We also found no correlation (R^2^ < 0.1, Pearson’s correlation) when we plotted exon differential expression against FUS binding indicating that, in general, R495X binding did not mediate substantial splicing changes (Supplementary Fig. S3). To account for potential intronic, instead of exonic, FUS binding that affects upstream or downstream exon selection, we measured R495X binding around up- and down-regulated exons (Fig. 3G). We found that the binding density was identical for up- and down-regulated exons and there was no preferential binding at any position around the exons. Again, we found a prominent density increase within the exons highlighting the mature mRNA binding preference of R495X. Collectively our results suggest that the majority of the observed gene expression and splicing changes are probably due to secondary effects and not directly related to FUS binding.

### R495X disturbs the translation efficiency of mitochondrial associated genes

**Figure 4:**
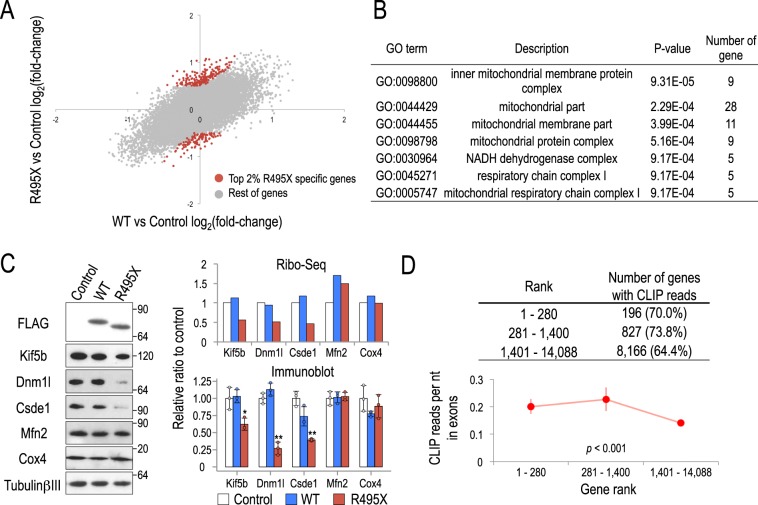
Differential gene translation analysis for WT- and R495X-expressing neurons. (**A**) Scatter plot of WT versus control and R495X versus control differential gene translation. The top 2% (280) differentially translated genes that are specific to R495X are shown in red. (**B**) Gene ontology enriched terms for the top 2% (280) differentially translated genes specific to R495X compared to the remaining genes. (**C**) Immunoblots of differentially translated genes in R495X-expressing neurons (left). Bar plots for gene translational levels quantified from Ribo-Seq (top right) and protein band intensities quantified from immunoblots (bottom right). Protein standard is shown on the right. Band intensities were normalized by TubulinβIII and the average value for control was set to 1. P-values in one-way ANOVA are, 0.01, < 0.0001, 0.001, 0.725 and 0.257 for Kif5b, Dnm1l, Csde1, Mfn2 and Cox4, respectively. ^*^p < 0.05, ^**^p < 0.01 to control in post Tukey’s HSD test. Error bar indicates standard deviation (N = 3). (**D**) Number of genes that are bound by R495X for gene sets of decreasing differential translation in R495X (top) and R495X binding density in gene exons for the same gene sets (bottom). Error bars indicate standard error. P-value, one-way ANOVA test.

To examine R495X effects on mRNA translation we analyzed differential translation between WT and control and R495X and control libraries. Similar to RNA-Seq, the control Ribo-Seq library was prepared from non-transduced neurons that express endogenous FUS. To select R495X-specific differentially translated genes we first identified genes with no difference between WT and control (−0.5 < log_2_(fold-change) <0.5), selected those with high differential translation between R495X and control (log_2_(fold-change) <−0.5 or >0.5) and ranked them by p-value. Out of the top 2% (280) differentially translated genes (Fig. 4A and Supplementary Table S16) only 33 (~12%) displayed significant expression differences indicating that differential translation cannot be attributed to changes in mRNA abundance. Interestingly, GO analysis revealed that the top differentially translated genes were associated with mitochondria function (Fig. 4B and Supplementary Table S17) raising the possibility that R495X-induced protein changes affect mitochondria.

To further test this, we used immunoblot to measure the protein levels of the mitochondria associated genes Kif5b, Dnm1l and Csde1 along with Mfn2 and Cox4 that are also mitochondria related but their translation was unaffected in R495X. We found that the protein levels of Kif5b (*p* < 0.05), Dnm1l (*p* < 0.01) and Csde1 (*p* < 0.01) were significantly reduced in R495X-expressing neurons compared to control and WT, consistent with the ribosome profiling results (Fig. 4C). As expected, Mfn2 and Cox4 showed no significant difference (*p* > 0.05) in any combination. Interestingly, we also found that R495X targeted a marginally higher percent and had a significantly higher binding density on the top ranked differentially translated genes than the lower ranked (Fig. 4D). Importantly, we also observed a similar protein level reduction of Csde1 and Dnm1l in neurons expressing substantially less R495X, indicating that this phenotype is not a result of abnormally high R495X expression (Supplementary Fig. S6). Collectively, our results indicate that R495X binds and changes the translation profile of a set of target genes, particularly related to mitochondria function.

### R495X reduces mitochondria size

**Figure 5:**
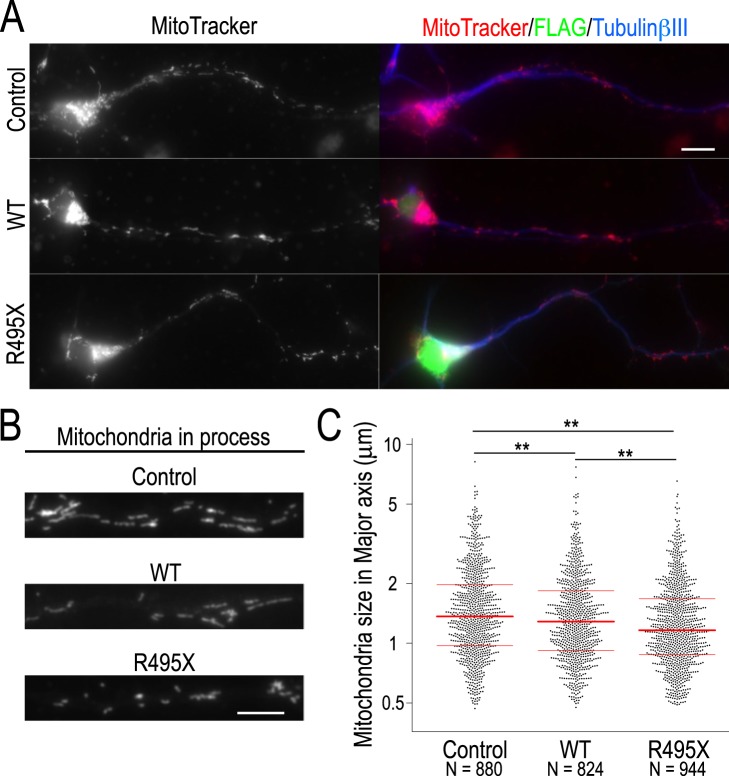
Mitochondria size for control, WT- and R495X-expressing neurons. (**A**) Images of mitochondria stained with MitoTracker (left) and merged images stained with anti-FLAG antibody (green), anti-TubulinβIII (blue) and MitoTracker (red) (right) for control (top), WT- (middle) and R495X-expressing neurons (bottom). Scale bar, 10 μm. (**B**) Magnified images of MitoTracker stained neuronal processes for control (top), WT- (middle) and R495X-expressing neurons (bottom). Scale bar, 5 μm. (**C**) Beeswarm plot for mitochondria major-axis length in control, WT- and R495X-expressing neurons. The number of analyzed mitochondria is indicated at the bottom. Red lines indicate the median, 25th and 75th percentiles. *p* < 0.0001, one-way ANOVA. ^**^p < 0.01, post Tukey’s HSD test.

We observed that Dnm1l translation, a known regulator of mitochondrial fission^28^, was significantly reduced in R495X-expressing neurons (Fig. 4C) raising the possibility that mitochondria morphology might be altered. To test this, we visualized mitochondria with MitoTracker staining and subjected them to immunostaining with anti-FLAG and anti-TubulinβIII antibodies. We found that, in all conditions, mitochondria were localized mainly in the cell soma but were also distributed in the neuronal processes (Fig. 5A). Measuring mitochondria size by quantifying their major axis length, we found that mitochondria that were localized in the processes of R495X-expressing neurons were smaller than those in control and WT-expressing neurons (Fig. 5B). The average length of the major mitochondrial axis was 1.63 ± 0.03 μm, 1.50 ± 0.03 μm and 1.38 ± 0.02 μm for control and WT- and R495X-expressing neurons respectively. We found the difference to be statistically significant for all combinations (Fig. 5C, *p* < 0.01). We observed a consistent result in neurons expressing lower levels of R495X, while no significant shortening of mitochondria was observed in neurons expressing lower levels of WT (Supplementary Fig. S7), suggesting that FUS overexpression results in mitochondria shortening and R495X significantly increases the effect.

### 4FL mutation in the RNA recognition motif of R495X alters its binding

**Figure 6:**
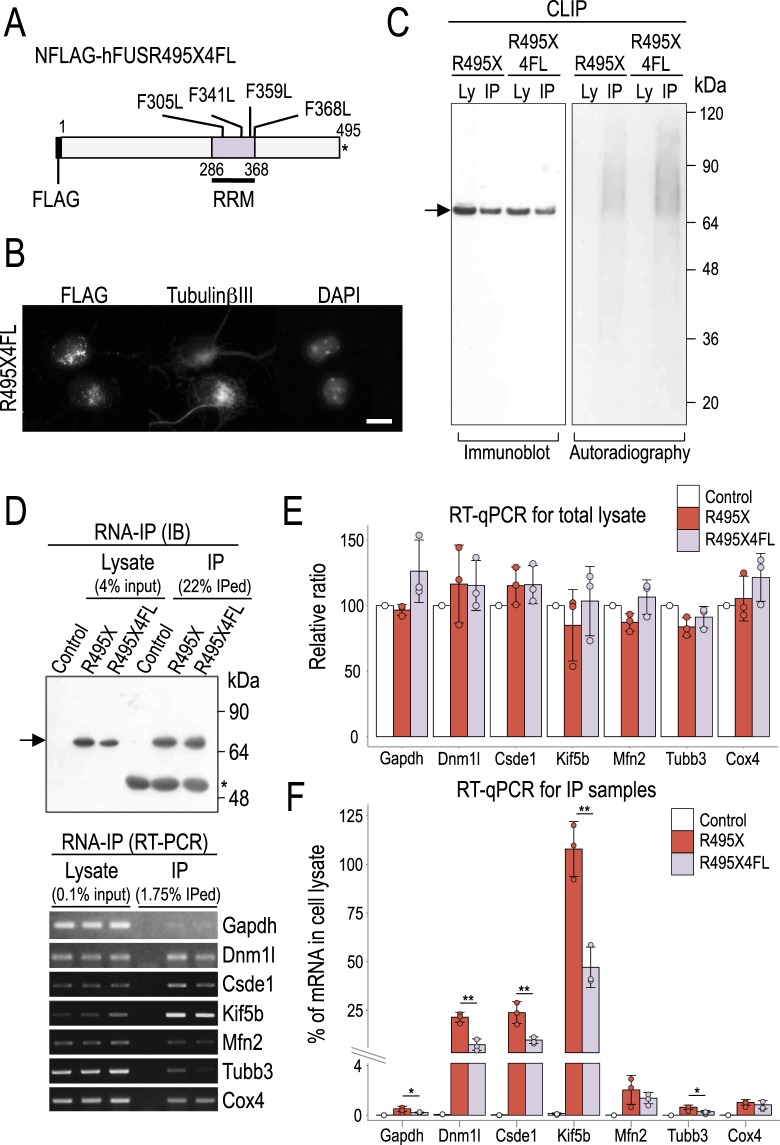
The RNA binding profile of R495X4FL mutant. (**A**) Schematic of the NFLAG-hFUSR495X4FL (R495X4FL) mutant structure. The 4 Phenylalanines at the indicated positions of the FUS RRM were substituted to Leucines. Asterisk indicates stop codon. (**B**) Immunostaining images for anti-FLAG (left), anti-TubulinβIII (middle) and DAPI stain (right) show the cellular localization of R495X4FL in neurons. Scale bar, 10 μm. (**C**) Cell lysate (Ly) and CLIP samples (IP) with a rabbit anti-FLAG polyclonal antibody from neurons expressing R495X or R495X4FL were analyzed by immunoblot with a mouse monoclonal anti-FLAG antibody (left panel) and autoradiography (right panel). Arrow indicates FLAG tagged proteins. The numbers at the right indicate protein standards. (**D**) Immunoblot of cell lysate and IP samples using anti-FLAG antibody (top). Arrow indicates FLAG tagged proteins. The numbers at the right indicate protein standards. Asterisk indicates IgG heavy chain. RT-PCR using RNA samples from cell lysate and IP samples for the genes indicated at the right (bottom). (**E**) Gene expression levels measured by qPCR from total RNA from cell lysate. Control expression was set to 100% and the relative gene expression was measured. Error bar indicates standard deviation (N = 3). No statistically significant difference was observed for any gene. P-values in one-way ANOVA are, 0.077, 0.574, 0.250, 0.579, 0.079, 0.051 and 0.243 for Gapdh, Dnm1l, Csde1, Kif5b, Mfn2, Tubb3 and Cox4, respectively. (**F**) Gene expression levels measured by qPCR for RNA from IP samples. mRNA abundance is measured as percent of input. Lower part corresponds to [0, 4]%, while upper part to [4, 125]%. P-values in one-way ANOVA are, 0.006, < 0.0001, 0.0004, < 0.0001, 0.035, 0.001 and 0.004 for Gapdh, Dnm1l, Csde1, Kif5b, Mfn2, Tubb3 and Cox4, respectively and ^*^p < 0.05 and ^**^p < 0.01 between R495X and R495X4FL in post Tukey’s HSD test. Error bar indicates standard deviation (N = 3).

To test the specificity of the observed effects with respect to the RNA binding of R495X we substituted the Phenylalanines at positions 305, 341, 359 and 368, within the FUS RRM, with Leucine (4FL). A previous report had shown that the introduction of 4FL in WT FUS reduces its RNA binding ability^29^. To test this, we prepared a construct coding for the amino-terminally FLAG tagged R495X4FL mutant (NFLAG-hR495X4FL, R495X4FL, Fig. 6A) and expressed it in neurons. Immunostaining revealed that R495X4FL localization was similar to R495X (Fig. 6B). Using CLIP to compare the RNA binding of R495X4FL to R495X, we found that, unexpectedly, both proteins showed indistinguishable results highlighted by a smear of bound RNA in the autoradiography, indicating that R495X4FL could efficiently bind RNA (Fig. 6C).

Previous reports have shown that FUS binds RNA through the Gly-rich, RGG1, ZnF and RGG2^30,31^ in addition to the RRM. Therefore, we hypothesized that while the binding ability of R495X4FL might be largely unaffected, its binding specificity might be disturbed. To test this, we performed RNA-immunoprecipitation (RNA-IP) for 5 mitochondria associated genes along with Gapdh and Tubb3. Both R495X4FL and R495X were specifically precipitated and RT-PCR products showed a specific band for each tested gene in the Lysate and IP samples (Fig. 6D). We used qPCR to quantify the relative abundance of PCR products setting the mRNA abundance of the control total cell lysate to 100%. We observed no significant difference between gene expression levels under any condition (*p* > 0.05, Fig. 6E), indicating that neither R495X4FL nor R495X affected the mRNA abundance of the tested genes. Then, we measured the mRNA IP abundance as a percent of mRNA abundance in total cell lysate. We observed that R495X4FL bound with significantly (*p* < 0.05) lower affinity than R495X on 5 out of 7 tested genes (Dnm1l, Csde1, Kif5b, Gapdh and Tubb3) and with marginally lower affinity on the other two (Mfn2 and Cox4) (Fig. 6F) indicating that 4FL altered the binding features of R495X.

### 4FL mutation partially rescues R495X-induced mitochondria shortening and neurotoxicity

**Figure 7:**
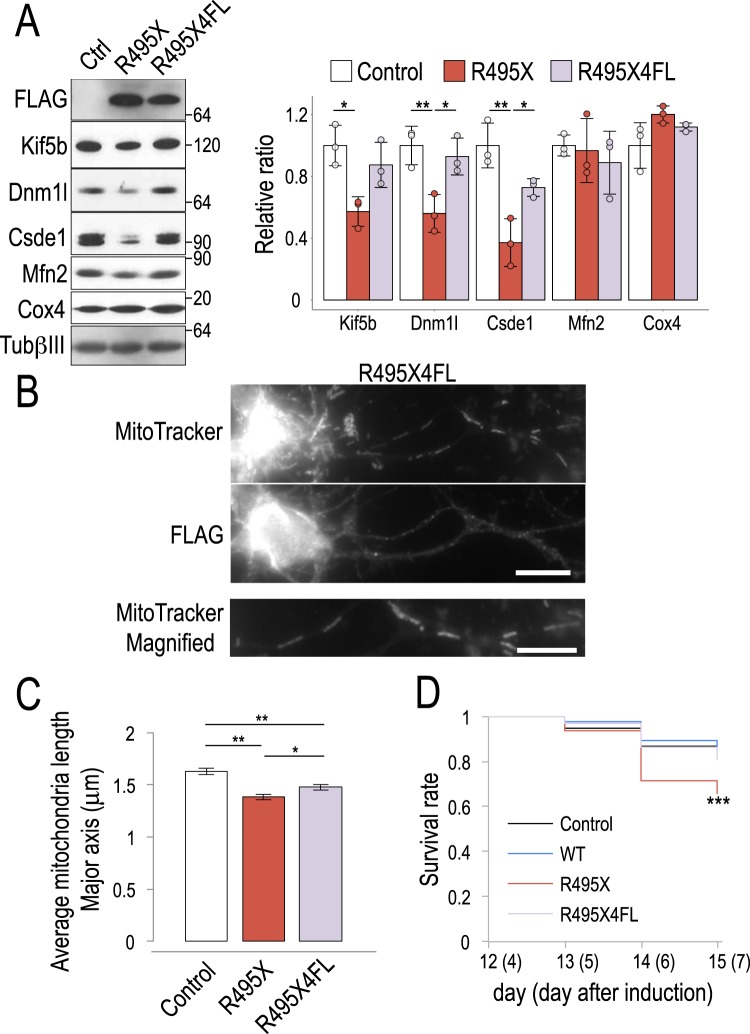
R495X4FL partially rescues R495X induced mitochondria shortening and neurotoxicity. (**A**) Immunoblots for control, R495X- and R495X4FL-expressing neurons (left) and bar plot for normalized protein band intensities (right). Average band intensity for control was set to 1. Protein standard is shown on the right. Error bars indicate standard deviation (N = 3). P-values in one-way ANOVA are, 0.015, 0.009, 0.003, 0.731 and 0.093 for Kif5b, Dnm1l, Csde1, Mfn2 and Cox4, respectively and ^*^p < 0.05 and ^**^p < 0.01 in post Tukey’s HSD test. (**B**) Staining images using MitoTracker (top) and anti-FLAG antibody (middle) with magnified images of MitoTracker stained neuronal processes (bottom) of an R495X4FL-expressing neuron. Scale bar, 10 μm (middle) and 5 μm (bottom). (**C**) Average mitochondria major-axis length for control, R495X- and R495X4FL-expressing neurons. 880 mitochondria from 10 cells expressing R495X4FL were analyzed. Data for control and R495X are copied from Fig. 5C. Error bars indicate standard error. P-value in one-way ANOVA is < 0.0001 and ^*^p < 0.05 and ^**^p < 0.01 in post Tukey’s HSD test. (**D**) Cell survival curve for control, WT-, R495X- and R495X4FL-expressing neurons. The number of surviving cells at days 13–15 are expressed as the ratio over those surviving at day 12 (y-axis). The numbers in parenthesis indicate the day after induction. ^***^p < 0.003, log-rank test.

To test whether the altered binding specificity of R495X4FL also changed the protein output of the affected genes, we prepared cell lysates from control, R495X- and R495X4FL-expressing neurons and analyzed them by immunoblot. We found that Dnm1l, Csde1 and Kif5b had no significant protein level changes in R495X4FL compared to control which indicates that 4FL reverted the observed protein effects induced by R495X (Fig. 7A). To test whether 4FL could also rescue the R495X-induced mitochondria size reduction in neuronal processes, we stained neurons expressing R495X4FL with MitoTracker and employed immunostaining with anti-FLAG antibody (Fig. 7B). Interestingly, we found very few short mitochondria compared to R495X-expressing neurons (Fig. 5B). Quantification of the mitochondria size revealed that, in R495X4FL-expressing neurons, mitochondria were significantly longer than R495X with an average major axis length of 1.48 ± 0.03 μm (Fig. 7C). The observed length was close to WT FUS but still smaller than the control, indicating that FUS abnormal binding, due to overexpression- or mutation-induced mislocalization, affects mitochondria size. Interestingly, similar to WT, R495X4FL at lower expression level also did not result in significant reduction of mitochondria size (Supplementary Fig. S7) further highlighting the importance of the RRM in the phenotype. To test whether cells would compensate for the reduced mitochondria size by increasing their number, we measured mitochondria in neuronal processes but found no significant difference (Supplementary Fig. S4).

Then, we wished to test the toxicity of R495X to neurons. We analyzed the survival rate for Control, WT-, R495X- and R495X4FL-expressing cells and found that R495X induced cell death at significantly higher level compared to control and WT (*p* < 0.003) indicating that the mutation has adverse consequences on neurons. Strikingly we found that R495X4FL showed reduced evidence of cell death indicating that abolishing the RRM-directed targeting specificity of FUS can rescue the observed neurotoxicity (Fig. 7D). Collectively, our results show that 4FL partially rescues mitochondria size and neurotoxicity highlighting the key role of the RRM and the interaction of FUS with target RNA.

## Discussion

FUS mutation R495X results in severe ALS phenotype. We used CLIP-Seq, RNA-Seq and Ribo-Seq to comprehensively evaluate the mutation outcome, in neurons. We set to identify its impact on the FUS targetome and on gene expression and translation simultaneously. We revealed key differences on RNA binding and showed that R495X binds on mature mRNAs in the cytoplasm, in contrast to WT that binds on precursor mRNAs. Our finding is corroborated by a previous report that showed that FUS R521G mutant also mislocalizes to the cytoplasm and has weak binding in gene introns^6^. To evaluate whether the abnormal binding of R495X affects mRNA expression we performed RNA-Seq in control, WT- and R495X-expressing neurons and showed that R495X has modest effects on target gene expression. Furthermore we found that there is no correlation between R495X binding and differential expression raising the possibility that any observed changes are mostly indirect effects. This is supported by reports that implicate FUS to pre-mRNA processing but not gene expression regulation^6,10–15^ and other studies that found minor expression changes between FUS mutants and WT^7,32–34^. Consistent with our results, reports using mice embryonic brain expressing NLS-deleted FUS (FUS ΔNLS) or HEK293 cells expressing R495X identified only few genes with more than 1.5-fold expression differences^32,33^. Other reports using spinal cord from R521C transgenic mice^7^ and P525L expressed motor neurons from gene edited iPS cells^34^ showed cell adhesion terms enriched in differentially expressed genes, similar to our results for WT but not R495X.

Interestingly, by measuring gene translation changes in control, WT- and R495X-expressing neurons we identified several genes with translation level changes specific to R495X. We found that these genes were highly targeted by R495X and the majority of them had no expression changes, indicating that R495X binding affects gene translation directly. Moreover, we found that the differentially translated genes are associated with mitochondrial functions and in R495X-expressing neurons mitochondria are significantly shorter than in control and WT-expressing neurons. Consistent with our results, it has been shown that genes targeted by R521G and R521H FUS mutants in HEK293 cells, are enriched for mitochondria related terms^6^. Mitochondria dysfunction has been linked to ALS pathology and several studies have demonstrated that mitochondria morphology is disturbed in neurons of ALS patients and ALS model animals^35–38^. Also, ALS associated FUS mutants such as R521L, R521G, R521H, R521C, P525L and R495Qfs527X have been shown to induce shortening and fragmentation of mitochondria^39–44^ and studies involving human WT FUS transgenic mice showed motor neuron loss^16^ coupled with abnormalities in mitochondrial vacuolation and a loss of synaptic vesicles^45^. Nevertheless, more studies in disease models maintaining physiological FUS expression levels would be needed to directly show association of mitochondria shortening to ALS pathology. While precise molecular mechanisms are still lacking, we found that genes with high translational efficiency were enriched in mitochondria-related GO terms (Supplementary Table S18), raising the possibility that R495X affects translational efficiency.

To assess the role of the R495X RNA binding activity on mitochondria shortening, we introduced 4FL mutation to perturb the FUS RRM and disrupt the interaction with RNA. We found that while R495X4FL could still bind RNA, its binding specificity was significantly diminished and it bound to mitochondria associated genes significantly less than R495X. Also, compared to control, the protein levels of R495X-targeted genes were significantly reduced in R495X but not when 4FL was introduced. This indicates that R495X can disturb the translation of non-physiologically targeted mRNAs and affect mitochondria morphology and potentially function. Finally, we find that the introduction of 4FL partially rescues mitochondria shortening and abrogates the observed neurotoxicity induced by R495X. This is in agreement with past studies indicating that 4FL suppresses toxicity in yeast^46^ and neurodegeneration in flies^29^. Notably, we find that, in contrast to WT and R495X4FL, even very low R495X expression levels are sufficient to trigger mitochondria size reduction. However, our study does not distinguish the transcriptional, translational and targetome effects at varying FUS expression levels and further studies will be required to specify the exact changes. Our results reveal key aspects of R495X effect on mitochondria shortening and neurotoxicity and importantly highlight the involvement of the FUS RRM interaction with RNA in the process.

## Materials and Methods

### Maintenance and differentiation of mouse embryonic stem (ES) cells

Mouse ES cells (E14Tg2A.4, RRID:MMRRC_015890-UCD) were maintained and differentiated into neurons as previously described^10^. Briefly, cells were maintained in gelatin-coated dish with ES cell medium supplied with 10% FCS (Hyclone, SH30071.03). For differentiation, cells were cultured with ADFNK medium for two days, then medium was replaced with ADFNK supplied with 5 μM Retinoic Acid and cultured for three days. After the culture, medium was exchanged with neuron medium (ADFNB supplied with 5 ng/mL GDNF). Formed embryonic bodies were dispersed into single neurons by Trypsinization and seeded onto poly-L-lysine (Sigma) and Laminin (Thermo) coated plates with fresh neuron medium.

### Preparation of plasmids and viral supernatant

Human FUS WT or R495X with amino-terminal FLAG tag were amplified by PCR with primers; forward, 5′- CCCGGGACTAGTCACCATGGACTACAAGGACGACGATGACAAAATGGCCTCAA-3′, and reverse for WT, 5′- GACGCGGCCGCCTACAGATCCTCTTCTGAGATGAGTTTTTGTTAATA-3′, or for R495X, 5′- TACGCGGCCGCTCAGAAGCCTCCACGGTCCC-3′. 1st cDNA was prepared from total RNA isolated from HEK293T cells with SuperScriptIII (Thermo) and used as template for amplifications. The PCR products were digested with SpeI/NotI and cloned into pEN_Tmcs vector (https://www.addgene.org/25751/). The obtained plasmids were recombinated into pSLIK-Neo (https://www.addgene.org/25735/) vector with LR ClonaseII (Thermo). To obtain lentivirus supernatant, pSLIK vectors were transfected into HEK293T cells with pMD2G (https://www.addgene.org/12259/) and psPAX2 (https://www.addgene.org/12260/) vectors using Lipofectamine2000 (Thermo) and the produced lentivirus was precipitated from conditioned media by a centrifugation at 100,000 × g for 90 min. The viral pellet was resuspended with 1/100 volume of input of neuron medium and stored at −80 °C until use.

### Library preparation

The dispersed neurons were infected with lentivirus encoding NFLAG-hFUSWT or NFLAG-hFUSR495X at day 7. One day later, cells were treated with neuron medium together with 1 μg/mL doxycycline (Chemicon) and two days later with 400 μg/mL Geneticin (Thermo). The cells were incubated until day 12.

#### CLIP-Seq

For CLIP-Seq library preparation, cells (1 × 10^7^ cells in a 10 cm plate) at day 12 were irradiated with 400 mJ/cm^2^ UV, and then collected by a centrifugation, 5,000 × g for 5 min and used for library preparation as previously described^47^. For immunoprecipitation, 40 μL of a rabbit polyclonal anti-DYKDDDDK Tag antibody (CellSignaling, #2368) per sample was used. Libraries were sequenced with Illumina HiSeq, 50 nt, single-end. For WT and R495X we sequenced ~33 and ~25 million reads, respectively.

#### RNA-Seq

Cells at day12 were treated with 1 mL Trizol (Thermo) to isolate total RNA following the manufacturer’s protocol. The Ribosomal RNA was depleted by Ribo-Zero Gold kit (Illumina), and then submitted to Hokkaido System Science Co., Ltd to prepare libraries and sequenced with Illumina HiSeq, 101 nt, paired-end. We sequenced 42 to 46 million reads per sample.

#### Ribo-Seq

Ribo-Seq libraries were prepared with cells (1 × 10^7^ in a 10 cm plate) at day 12 as previously described^48^ with some modifications. Briefly, cells were washed with PBS and collected with lysis buffer (20 mM Tris pH7.4, 150 mM NaCl, 5 mM MgCl_2_, 1 mM DTT, 1% TritonX-100 and 25 U/mL Turbo DNase I (Thermo, AM2238)) and incubated on ice for 10 min. The cell lysate was passed through 27 G needle 10 times, and supernatant was collected by a centrifugation, 10,000 × g for 10 min. Obtained cell lysate was incubated with 1,000 U of RNaseI (Thermo, AM2294) for 45 min at room temperature. The reaction was stopped by addition of 14 μL of SUPERase.In (Thermo, AM2694). Monosomes were collected by an ultracentrifugation with 1 M Sucrose cushion, 200,000 × g for 4 hrs in TLA-100.3 (Beckman). The ribosome protected mRNA fragments (RPFs) were extracted from monosome pellet with Trizol. Ribosomal RNA was depleted with Ribo-Zero Gold kit (Illumina) and RPFs were fractionated with 15% denaturing PAGE to obtain a range from 25 to 35 nt. RPFs were extracted from gel, treated with alkaline phosphatase (Roche) and ligated to the 3′ linker conjugated with biotin^47^. After the 3′ linker ligation, T4 polynucleotide kinase (NEB) was directly added to the ligation reaction and incubated at 37 °C for 20 min followed by incubation at 65 °C for 20 min with an addition of EDTA at final 10 mM. Samples were subjected to library preparation as described for CLIP-Seq. Libraries were sequenced with Illumina HiSeq, 50 nt, single-end. We sequenced 27 to 29 million reads per sample.

#### CLIP-Seq and Ribo-Seq data preprocessing

The adaptor sequences were trimmed from the reads using the cutadapt software. The adaptor AGGGAGGACGATGCGG was trimmed from the 5′-end of reads and GTGTCAGTCACTTCCAGCGGTCGTATGCCGTCTTCTGCTTG was trimmed from the 3′-end allowing for 0.25 error rate. Only reads longer than 15-nt after adaptor trimming were retained. The reads were aligned to the mouse genome (mm9) using the STAR aligner (v2.4.1c)^49^ and the following parameters –outFilterMultimapScoreRange 0 –alignIntronMax 50000 –outFilterIntronMotifs RemoveNoncanonicalUnannotated –outFilterMatchNmin 15 –outFilterMatchNminOverLread 0.9 –sjdbOverhang 100. Reads mapping to areas annotated by RepeatMasker as ribosomal RNAs or tRNAs were excluded from further analysis. Aligned reads were loaded into an SQLite3 database for further processing with CLIPSeqTools^27^ and were annotated with information whether they overlap with elements from RepeatMasker (downloaded from UCSC) and genes (from UCSC gene model annotation file). **RNA-Seq data preprocessing**. The adaptor sequences were trimmed from the reads using the cutadapt software in paired-end mode. The adaptor AGATCGGAAGAGCACACGTCTGAACTCCAGTCAC was trimmed from the 3′-end of mate 1 reads and AGATCGGAAGAGCGTCGTGTAGGGAAAGAGTGTA was trimmed from the 3′-end of mate 2 reads allowing for 0.25 error rate. Paired-end alignment was performed using STAR with the same parameters as for CLIP and Ribo-Seq. Downstream preprocessing steps were performed as for CLIP and Ribo-Seq. **Gene read count normalization**. Read count normalization was performed from the raw counts using the DESeq2^50^ package in R.

### Gene ontology enrichment analysis

For identification of GO terms enriched in differentially expressed or translated genes, we compared differentially changed genes (up- and down-regulated) against all other expressed genes using GOrilla (http://cbl-gorilla.cs.technion.ac.il). For identification of GO terms enriched in highly translated genes, we first calculated gene translation efficiency by normalizing Ribo-Seq with RNA-Seq for control samples as in^51^. We then compared the top 20% to all other genes (13,542 genes).

### Immunoblot

Immunoblot was performed as previously described^10^ using the following antibodies; mouse monoclonal anti-FLAG (Sigma, M2, 1/10,000), anti-Kif5b (Chemicon, MAB1614, 1/5,000), anti-Dnm1l (BD, 611112, 1/10,000) antibodies and Tuj1 (1/10,000), and rabbit polyclonal anti-FUS N-terminal (Bethyl, A300-302A, 1/10,000), anti-FUS C-terminal (raised against 510–523 a.a. of human FUS, 1/10,000), anti-Csde1 (Bethyl, A303–160A-T, 1/2,000), anti-Cox4 (Cell Signaling, #4844, 1/10,000) and anti-Mfn2 (Cell Signaling, #9482, 1/2,000) antibodies. Protein band quantification was performed with ImageJ (NIH). Uncropped images are shown in Supplementary Fig. S5.

### Immunofluorescence and image analysis

5 × 10^4^ cells of neurons on 8-well chamber (Thermo, #155409) were fixed with 3.7% paraformaldehyde in PBS for 10 min, then treated with 0.1% triton X-100 and 1% BSA in PBS for 10 min followed by three times wash with PBS. The cells were blocked with 3% BSA in PBS for 10 min. Primary antibody was diluted in 1% BSA in PBS and incubated with the fixed cells for O/N at 4 °C. After three times wash with PBS, cells were incubated with secondary antibody for 1 hr at room temperature. Then, the cells were washed three times with PBS and mounted with ProLong Gold with DAPI (Thermo, P36931). The used primary antibodies were mouse monoclonal anti-FLAG (Sigma, M2, 1/10,000) and rabbit polyclonal anti-TubulinβIII (Cell Signaling, #5568, 1/5,000). The secondary antibodies used were donkey anti-mouse IgG Alexa Fluor 488, goat anti-rabbit IgG Alexa Fluor 546 and goat anti-rabbit IgG Alexa Fluor 647 antibodies (Thermo). To stain mitochondria, live cells on plate were incubated with MitoTracker Red CMXRos (Thermo, M7512, 10 nM) for 45 min and then fixed. Images were obtained under a fluorescence microscope. To quantify the major mitochondrial axis length, the obtained images were processed with macro, Mitochondrial Morphology^52^, in ImageJ. To quantify the signal intensities in subcellular fractions, we first isolated 20 μm x 20 μm squares from the cell soma region. The signal that co-localized with DAPI staining was defined as nuclear, while the signal that co-localized with anti-TubulinβIII antibody was defined as cytoplasmic. The intensity was quantified with ImageJ and represented as the ratio of nuclear to cell soma. We counted the fraction of cells stained with anti-TubulinβIII and DAPI over those stained with DAPI alone to quantify the neuronal content. Similarly, we also counted the fraction of cells stained with anti-FLAG in the same areas described above to quantify the fraction of cells expressing exogenous proteins.

### RT-qPCR

Total RNA was isolated from neurons expressing WT, R495X, and control cells using Trizol, and then treated with DNaseI (Promega, M6101) for 30 min at 37 °C, extracted with Acid Phenol Chloroform (Ambion, AM9722) and recovered by ethanol precipitation. 250 ng of total RNA was subject for reverse transcription using SuperScriptIII (Thermo). 0.5 μL of 1st cDNA prepared above was used as template for qPCR using THUNDERBIRD SYBR qPCR Mix (TOYOBO) with specific primer sets (forward, reverse); Foxj1 (5′-GAACTGCCTCCCAAGACAGG-3′, 5′-CCTTGGGCTTGAGGGAACAT-3′), Zfp608 (5′-CTCCGGTGGTCACCTCTATG-3′, 5′-GACCGAGTCCGAACCAAAAG-3′), Nefm (5′-CAAGTGGGAAATGGCTCGTC-3′, 5′-TGTCGGTGTGTGTACAGAGG-3′), Slc17a6 (5′-AGGATATATCGCATCGCGGC-3′, 5′-CCATAATGCACTCTGGCTGC-3′), Apba1 (5′-TACAGTGACCTGCTCAACACC-3′, 5′-CTTCACTCGGGACTGGTTCTT-3′), Inppl1 (5′-AGTTCCTGACCTTCTTGTCCC-3′, 5′-TGCTACTCCTTCCTGCTCAGA-3′), mKIAA4052 (5′-GTAGCGTCCTCTCTGCACTCT-3′, 5′-GGACCGAGAGGAGATTTCACTG-3′), Zfp62 (5′- GTGAAGAAACTGCTGTACAAAGA-3′, 5′-GAGCAGGGTGTACGTTTCCA-3′), Zfp60 (5′-CAGTGGTGGGACGTTGCATT-3′, 5′-GCCATTACTAACACTGACGCC-3′), Islr2 (5′-ACAGATGCCAGAAGCTCCCT-3′, 5′-TGACCACTCCTAGCAAAGCC-3′), Enc1 (5′-GGAACAGAGACGCCCTTCGT-3′, 5′-TTCAGGCCACCACTGAACAT-3′), Pgm2l1 (5′-TTCTGTGTGGAACCTCCGTG-3′, 5′-TGGCTGGCTGCTATCATATCC-3′), Tubb3 (5′- GCGCCTTTGGACACCTATTC-3′, 5′-CTCCACTCATGGTGGCAGAC-3′), Csde1 (5′- TGGCCTACAACATCACACCC-3′, 5′- TATCTGGTCCCCTTGGCTGA-3′), Mfn2 (5′- TGGTCTCCATGGTTACTGGC-3′, 5′- GCGTATTCCACAAACTGGCG-3′), Dnm1l (5′- AACACGATTGAAGGAACCGC-3′, 5′- CTGGGCTCTTCTAGACGCTT-3′), Cox4 (5′- CAGGGGCACCAATGAATGGA-3′, 5′- ATAGTCCCACTTGGCGGAGA-3′), Kif5b (5′- GGAGGCAAGCAGTCGTAAAC-3′, 5′- AAACAGGGCCGCAGTTGTAA-3′) and Gapdh (5′- CAATGTGTCCGTCGTGGATCT-3′, 5′- GTCCTCAGTGTAGCCCAAGAT-3′). qPCR reaction was carried out using Mx3005P qPCR System (Agilent Technologies). The results were analyzed as previously described^10^.

### RNA-immunoprecipitation

Cells expressing R495X, R495X4FL or control were lysed with 100 μL of RSB200 (20 mM Tris pH 7.4, 200 mM NaCl and 2.5 mM MgCl_2_) supplied with 0.5% NP40 on ice for 20 min. Cell lysate was cleared by a centrifugation at 12,000 × g for 3 min. 4 μL of cell lysate was used for immunoblot and 20 μL of lysate was mixed with 100 μL of water and 380 μL of Trizol-LS (Thermo) for total RNA isolation. 70 μL of cell lysate was precleared with 5 μL of Protein G Dynabeads (Thermo) in 170 μL buffer in total for 30 min by rotation at 4 °C. For IP, 10 μL of Protein G Dynabeads was washed twice with RSB200 supplied with 0.5% NP40 and incubated with 1 μL of anti-FLAG antibody (M2, Sigma, F1804) for 1 hr by rotation at 4 °C. The beads were washed three times with buffer, mixed with precleared cell lysate and rotated for 3 hrs at 4 °C. After incubation, beads were washed five times with buffer and saved 8 μL of 35 μL suspension in buffer for immunoblot. The rest of the immunoprecipitated sample was resuspended in 500 μL of Trizol (Thermo). Total and IP RNA was isolated with Trizol. 250 ng of total RNA and RNA from IP were used for reverse transcription with SuperScriptIII. We confirmed that no non-specific RNA binding to beads was observed with our method using a normal mouse IgG (Supplementary Fig. S8).

### Cell survival curve

Neurons grown on 96-well plate (5 × 10^4^/well) were infected with lentivirus at day 0. Medium was replaced every day until day 7 after induction with 100 μL of fresh medium supplied with DOX (1 μg/mL final). The cell positions were recorded from day 4 to day 7 using a microscope. Stacked images from day 4 to 7 were aligned by the StackReg ImageJ (NIH) plug-in. We chased cells by TrackMate in ImageJ and analyzed the obtained results by the “survival” package in R, in which we tested statistical significance by log-rank test. We analyzed 2324, 3207, 1995 and 1903 cells for Control, WT-, R495X- and R495X4FL-expressing neurons, respectively.

### Statistical analysis

For all of statistical analyses, One-Way ANOVA and Tukey HSD Test was applied (http://vassarstats.net/anova1u.html) unless otherwise noted. Statistical significance was concluded for p-values < 0.05.

## Electronic supplementary material

Supplementary figures

Supplementary Tables S1 to S18

## Data Availability

The datasets generated in the current study are available in the Sequence Read Archive (SRA) under project ID: GSE106386
