# Supplementary material for: Amyotrophic Lateral Sclerosis associated FUS mutation shortens mitochondria and induces neurotoxicity
Source: Sci Rep. 2018 Oct 22;8:15575. doi: 10.1038/s41598-018-33964-0 (PMC6197261; doi:10.1038/s41598-018-33964-0)
Supplement: Supplementary file 1 — Supplementary figures [file 41598_2018_33964_MOESM1_ESM.pdf]

Supplementary Figures

Amyotrophic Lateral Sclerosis associated FUS mutation shortens mitochondria and induces neurotoxicity

Tadashi Nakaya[1]*, Manolis Maragkakis[2,3,4]*

[1]Laboratory of Neuroscience, Graduate School of Pharmaceutical Sciences, Hokkaido University, Sapporo, 060-0812, Japan, [2]Department of Pathology and Laboratory Medicine, Division of Neuropathology; [3]Institute for Translational Medicine and Therapeutics; [4]Penn Medicine Translational Neuroscience Center, Perelman School of Medicine, University of Pennsylvania, Philadelphia, Pennsylvania 19104, USA

Supplementary Figure S1. Immunostaining results in larger fields than Fig. 1B.

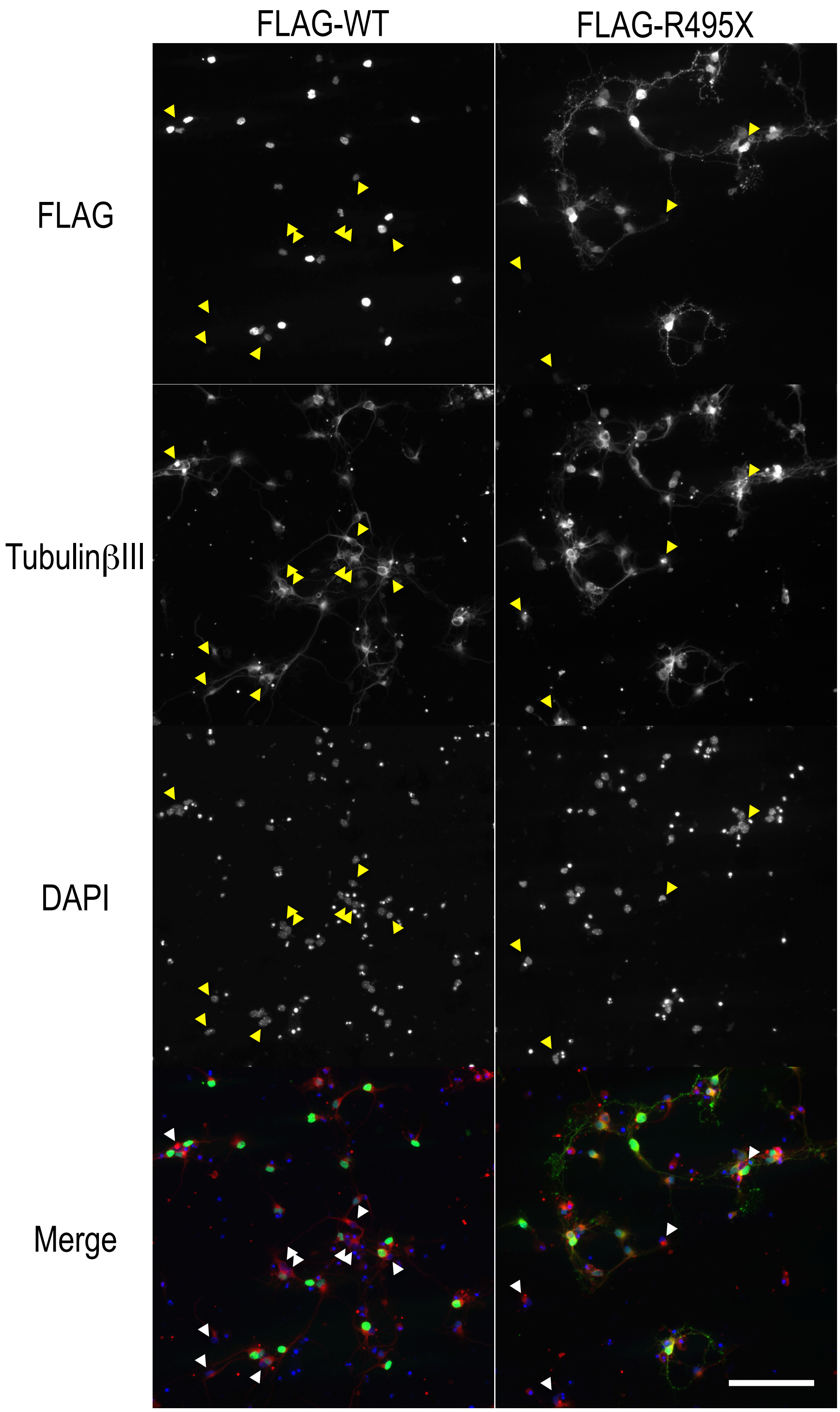

Images of anti-FLAG (top) and anti-TubulinβIII (second) antibody staining along with DAPI staining (third) for FLAG-WT (left) and FLAG-R495X (right) expressing cells. Bottom panels represent merged images (green, anti-FLAG, red, anti-TubulinβIII and blue, DAPI). Scale bar. 100 μm. Yellow and white arrows indicate cells positive to anti-TubulinβIII antibody staining, but negative to anti-FLAG antibody staining.

Supplementary Figure S2. Library preparation for CLIP-seq, RNA-seq and Ribo-seq

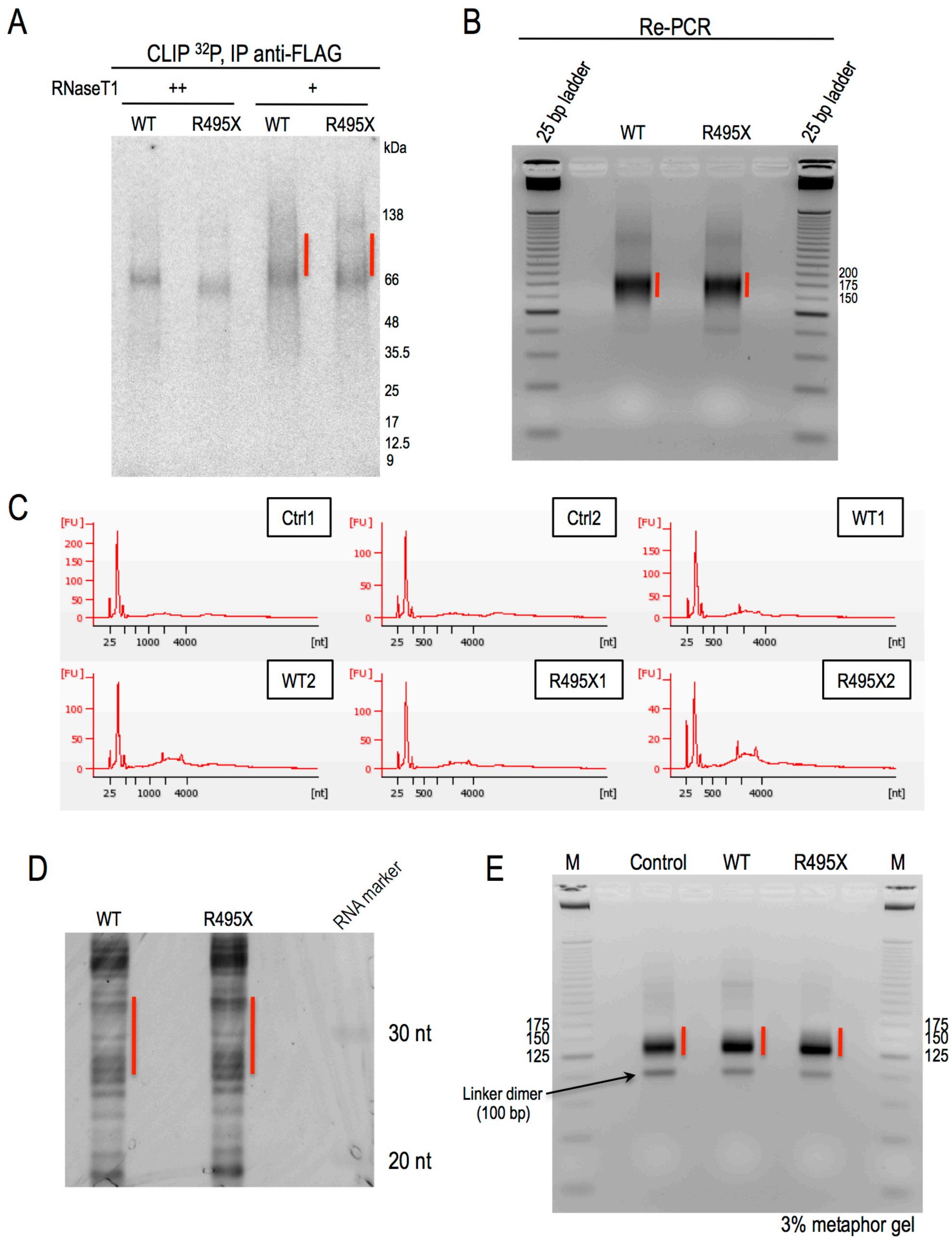

A. Gel image of ^{32}P labeled WT and R495X used for CLIP-Seq. Samples were prepared with two different amounts of RNaseT1 (++; high and + low concentration). Red bars indicate the cut out positions.

B. Gel image of Re-PCR for CLIP library preparation. Bands indicated by red bars were used for sequencing.

C. Results of QC of total RNA samples used for RNA-Seq.

D. Gel image of ribosome protected fragments in 15% denaturing PAGE from WT- and R495X-expressing neurons. Gel was stained with SYBR gold (Thermo, S11494). Bands indicated by red bars were used for library preparation.

E. Gel image of Re-PCR for Ribo-Seq library preparation. Bands indicated by red bars were used for sequence.

Supplementary Figure S3. Scatter plot for FUS binding versus exon expression fold-change levels for R495X. Color indicates gene density with yellow and blue indicating higher and lower values respectively.

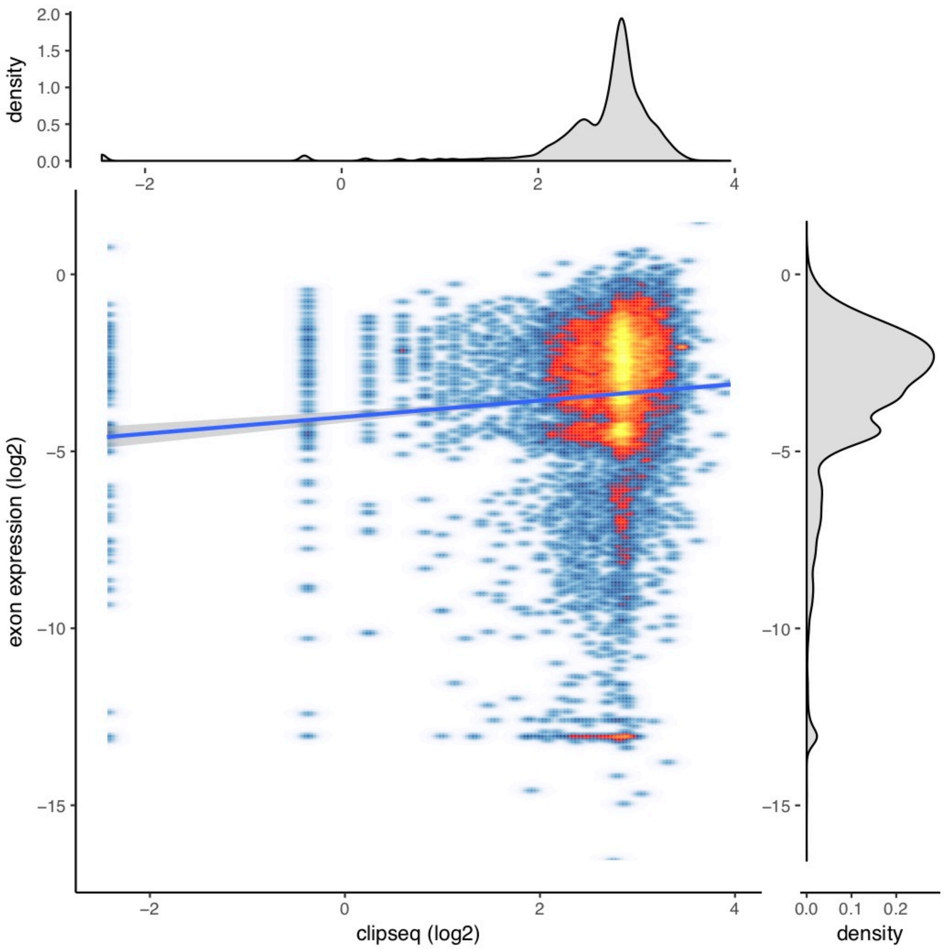

<u>Supplementary Figure S4. Number of mitochondria in 50 μm processes of WT-, R495X- and R495X4FL-expressing neurons or Control neurons.</u>

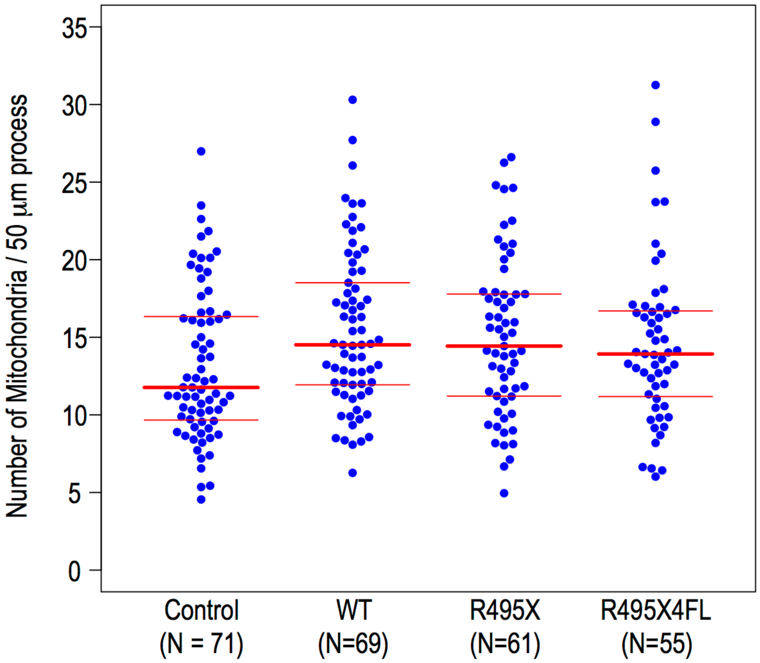

Beeswarm plot for the numbers of mitochondria per 50 μm processes of control, WT-, R495X and R495X4FL-expressing neurons. The number of analyzed processes is indicated at the bottom. Red lines indicate median, 75th and 25th percentiles. We found that Control, WT-, R495X- and R495X4FL-expressing cells have a median of 11.77, 14.51, 14.43 and 13.92 mitochondria and a standard deviation of 4.88, 5.14, 5.17 and 5.29, respectively, per 50 μm process. One-way ANOVA showed no significant difference ($p = 0.055$) between the conditions.

<u>**Supplementary Figure S5. Uncropped blot and gel images**</u>

For Fig. 1F. Numbers, 1, 2 and 3 at top indicates lanes for Control, WT and R495X, respectively.

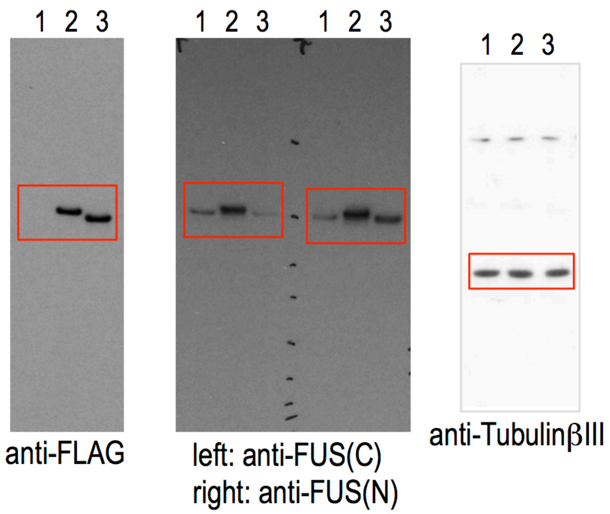

For Fig. 4C. We indicate % of gel used for analysis at bottom. We used cut membranes labeled with “upper” and “lower” for detection. Numbers at top indicates lanes for Control (1, 4, 7), WT (2, 5, 8) and R495X (3, 6, 9).

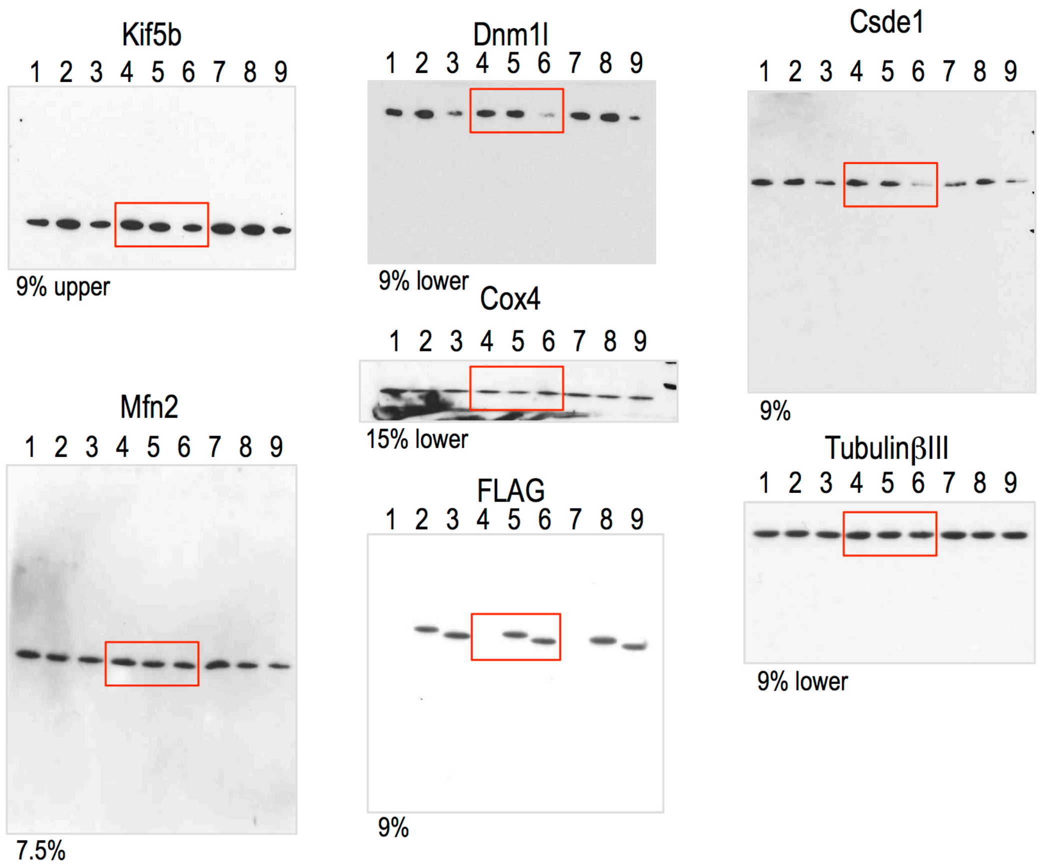

For Fig. 6D, IB. Numbers 1 to 6 at top indicates lanes for Control lysate, R495X lysate, R495X4FL lysate, Control IP, R495X IP and R495X4FL IP, respectively.

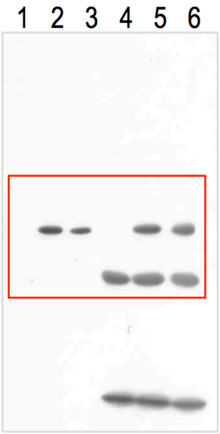

For Fig. 6D, RT-PCR. Numbers 1 to 6 at top indicates lanes for Control lysate, R495X lysate, R495X4FL lysate, Control IP, R495X IP and R495X4FL IP, respectively.

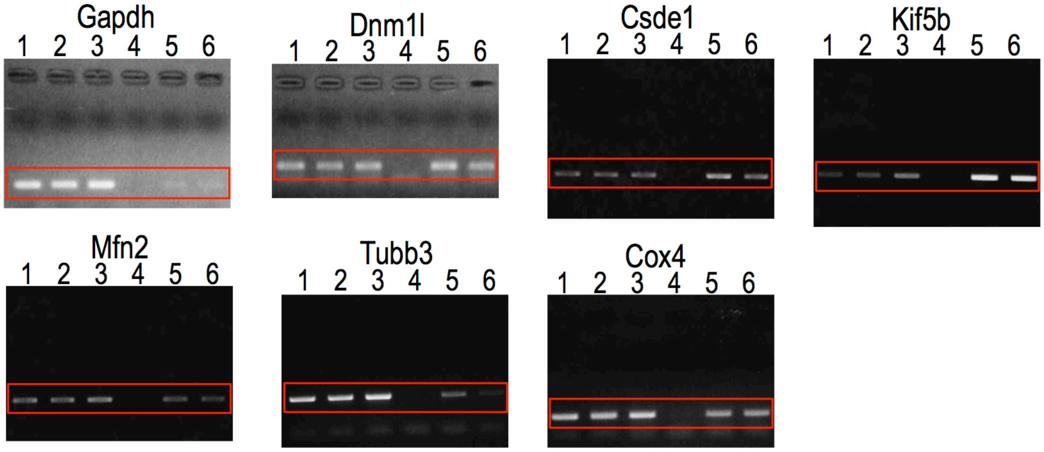

For Fig. 7A. We indicate % of gel used for analysis at bottom. We used cut membranes labeled with "upper" and "lower" for detection. Numbers at top indicates lanes for Control (1, 4, 7), R495X (2, 5, 8) and R495X4FL (3, 6, 9).

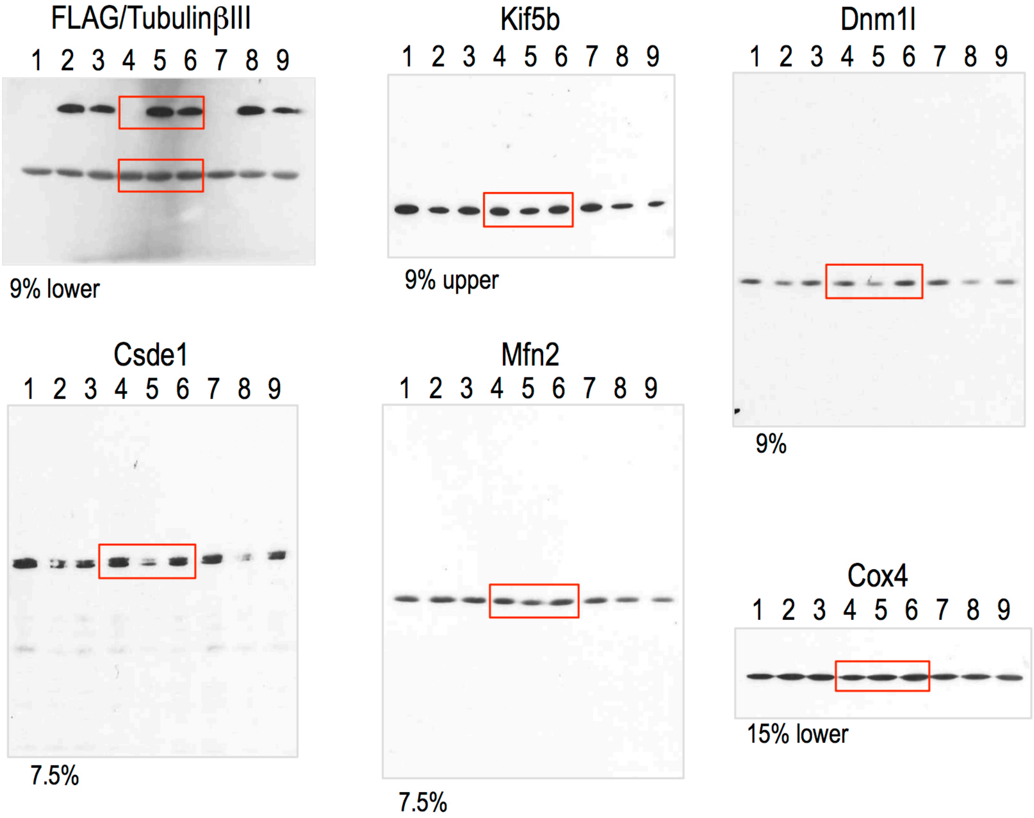

Uncropped gel images for Supplementary Fig. S6.

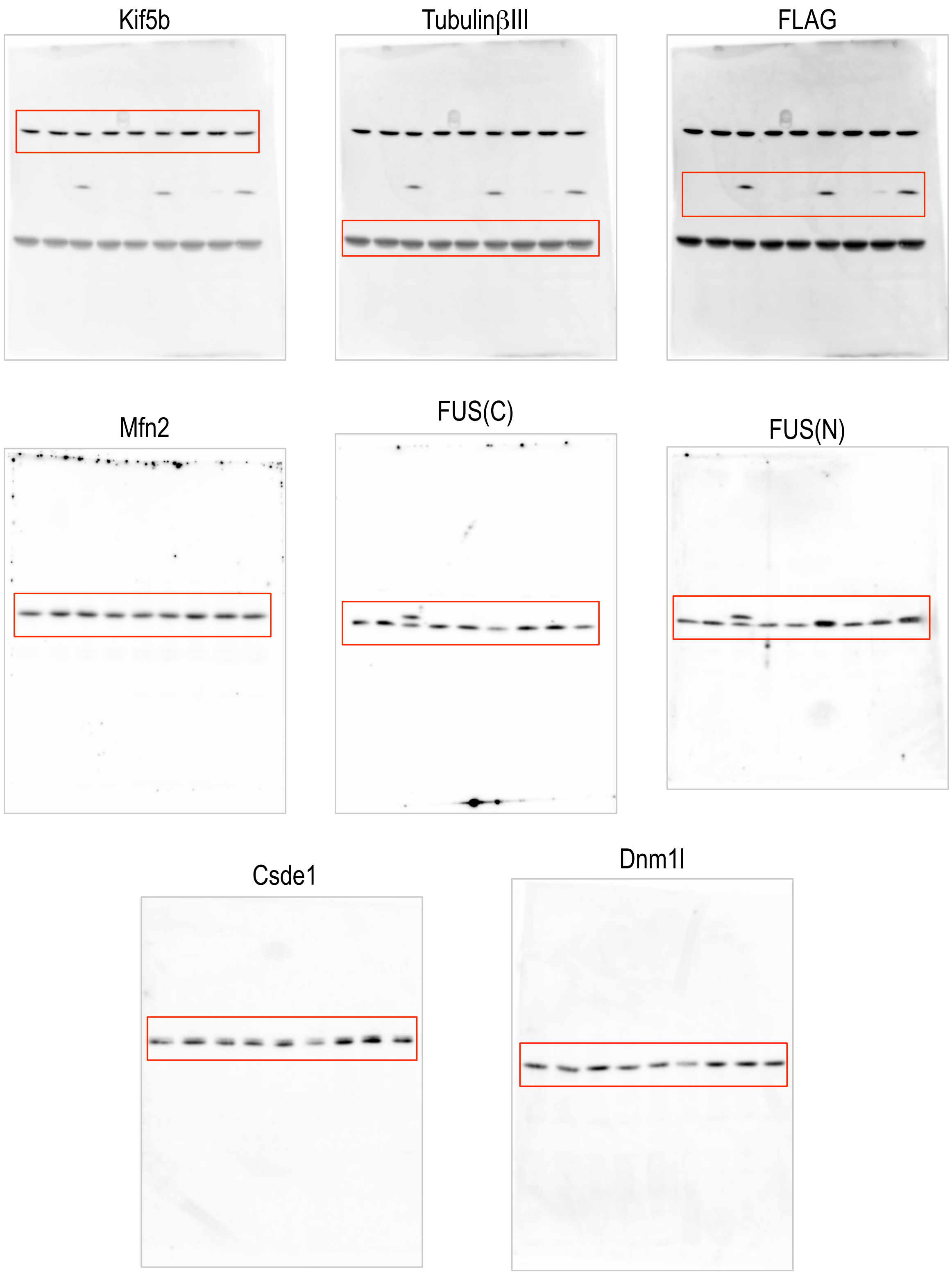

Uncropped gel images for Supplementary Fig. S8.

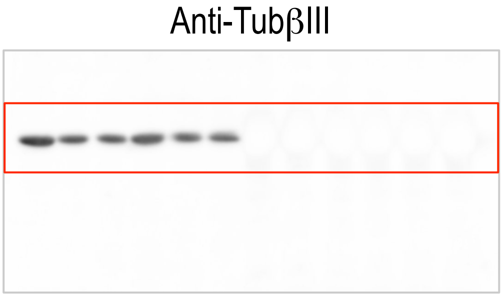

Supplementary Figure S6. Protein expression of mitochondrial genes in neurons expressing low levels of WT, R495X and R495X4FL.

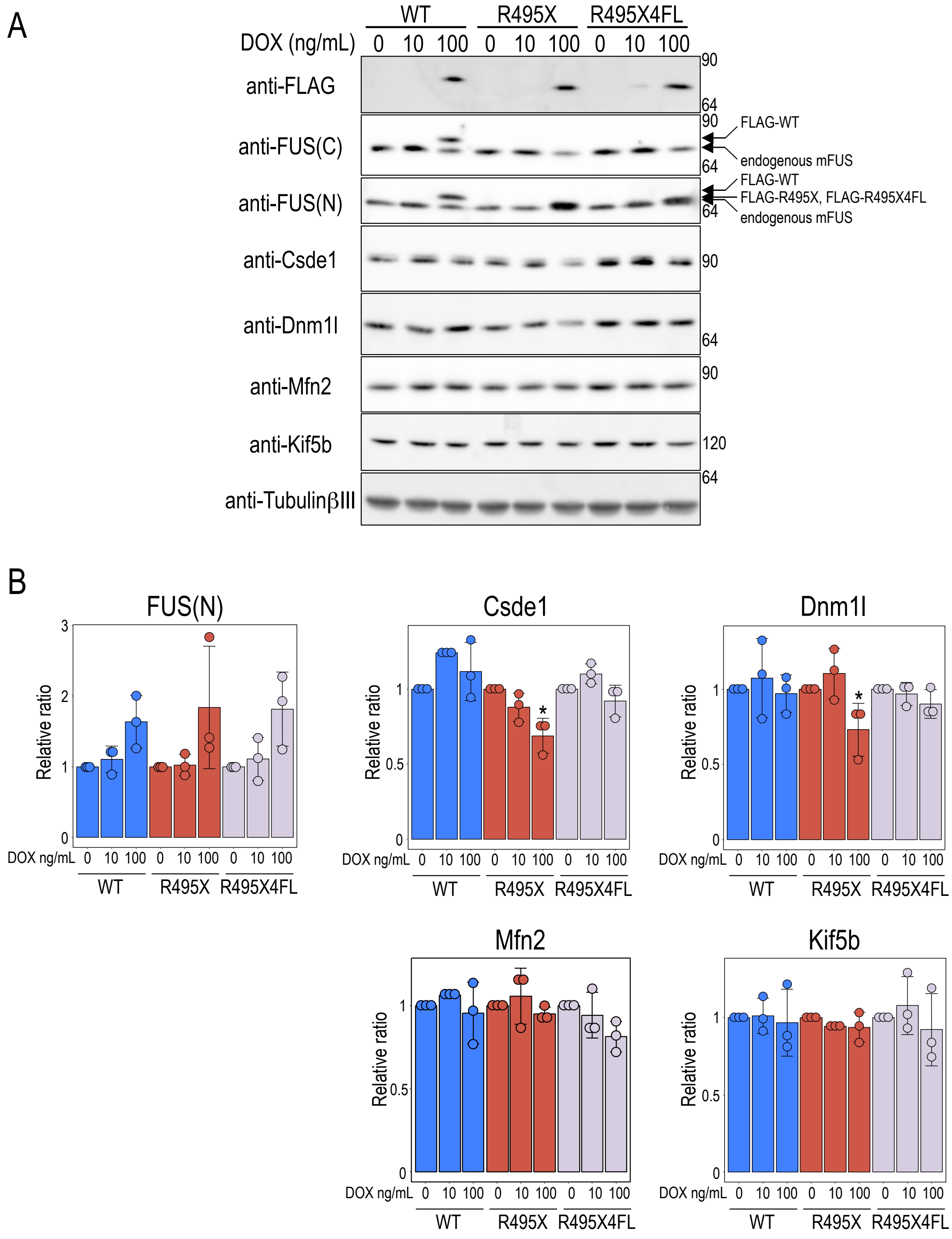

A. Immunoblots for neurons expressing lower WT, R495X or R495X4FL. Neurons infected with lentivirus coding FLAG-WT, FLAG-R495X or FLAG-R495X4FL were treated with DOX at indicated

concentration and 4 days after, cells were lyzed with RIPA buffer and analyzed by immunoblot using antibodies indicated at left. Numbers at right side indicate protein standards. Arrows at right indicate the positions of endogenous and exogenous FUS proteins. Uncropped gel images are shown in Supplementary Fig. S4

B. Bar plots for normalized protein band intensities. Protein band intensities in the results of immunoblot were normalized with the values of TubulinβIII and summarized in graphs. Samples without DOX in each condition were set to 1.0. Error bars indicate standard deviation (N=3). Statistical significance was obtained by one-way ANOVA following post-hoc Tukey HSD test. * < 0.05.

Supplementary Figure S7. Analysis of mitochondria size in neurons expressing low levels of WT, R495X and R495X4FL.

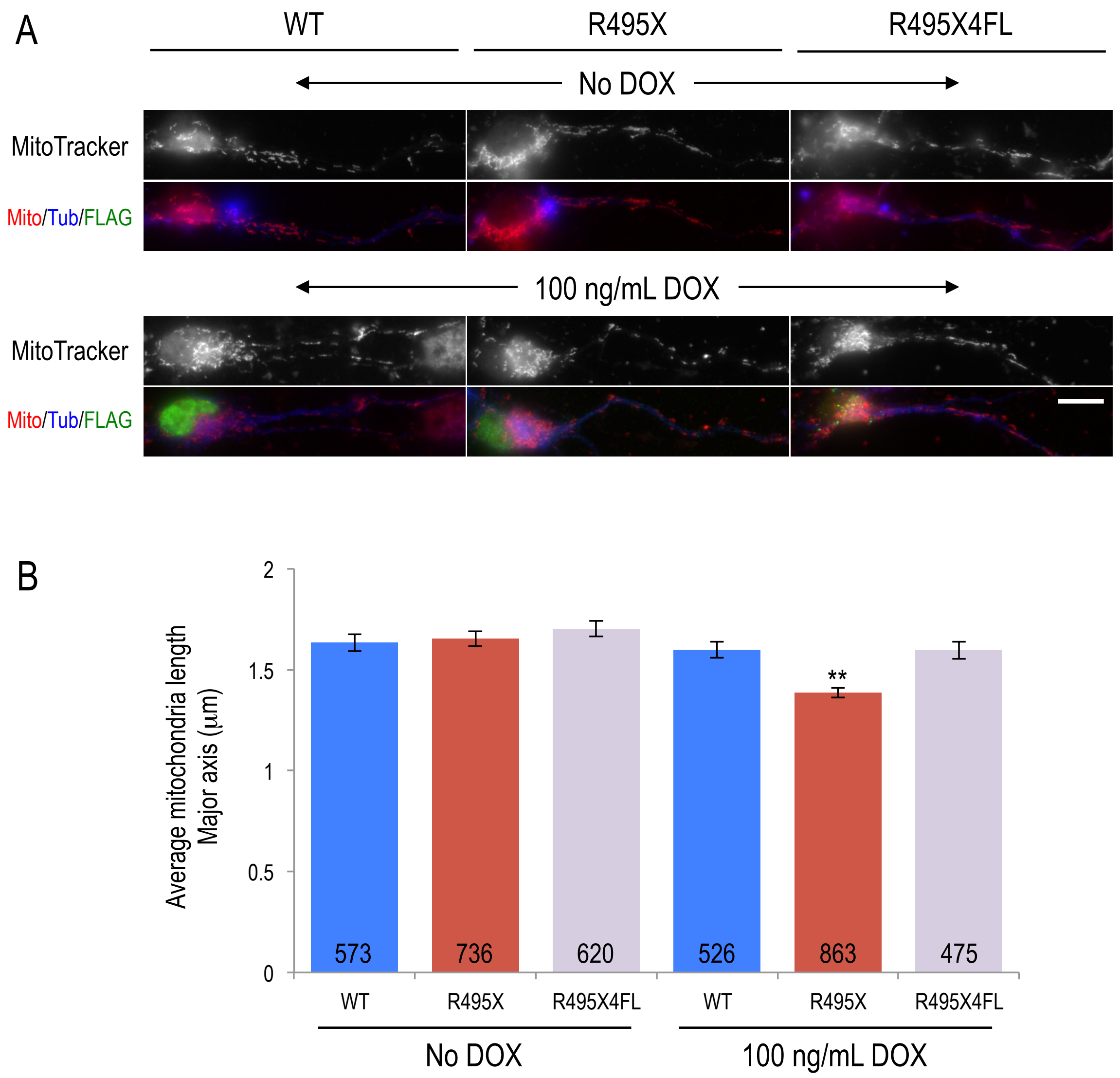

A. Representative images of immunofluorescence of neurons expressing WT (left), R495X (middle) and R495X4FL (right) at low levels. Immunofluorescence images of neurons untreated (No DOX) and treated with 100 ng/mL DOX are shown in upper and lower two rows, respectively. Scale bar, 10 µm. Other captions are same as in Fig. 5A.

B. Average mitochondria major axis length in neurons expressing WT, R495X and R495X4FL at low levels. Bar plots indicate the average size of mitochondria major axis in neurons infected lentivirus coding FLAG-WT, FLAG-R495X or FLAG-R495X4FL untreated (No DOX) and treated with 100 ng/mL DOX indicated at bottom. Numbers in columns indicate the number of mitochondria analyzed. Error bars indicate

standard error. Statistical significance was obtained by one-way ANOVA following post-hoc Tukey HSD test. ** < 0.01.

<u>Supplementary Figure S8.</u> RNA-IP analysis using a normal mouse IgG.

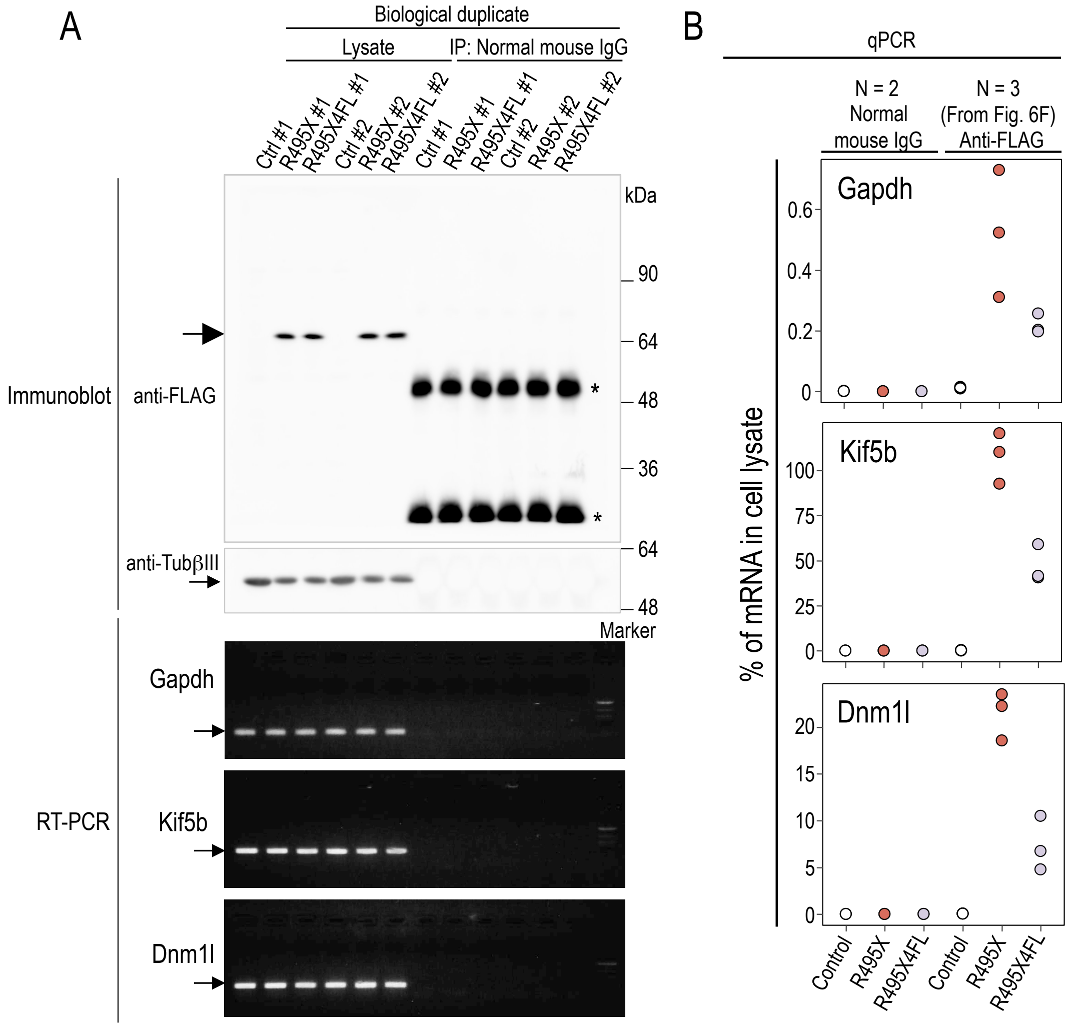

A. Result of RNA-IP using a normal mouse IgG for Control, R495X- and R495X4FL-expressing neurons. We performed same procedure described in the method section using a normal mouse IgG (SantaCruz biotechnology, sc-2025) instead of a mouse monoclonal anti-FLAG antibody. We used biological duplicate (#1 and #2) to validate the reproducibility. Top panel indicates the result of immunoblot using anti-FLAG antibody for cell lysate (left six lanes) and IP (right six lanes) samples. Analyzed samples are indicated at top. An arrow at left side indicates the position of R495X and R495X4FL. Asterisks indicate IgG heavy and light chains. Protein standards are indicated at right side. Second panel represents protein bands detected by an anti-TubulinβIII antibody (rabbit polyclonal). Three lower panels show the results of RT-PCR using RNA samples extracted from cell lysates (left six lanes) and IP (right six lanes) samples. Right end shows DNA marker. Arrows indicate the position of specific DNA bands.

B. Quantification of PCR products by qPCR. We applied same procedure described in the method section to calculate the recovery of specific PCR products in IP samples. Left three indicate the results using samples in A, while right three results are adapted from Fig. 6F. Each point indicates the result of single sample. We analyzed for Gapdh (top), Kif5b (middle) and Dnm1l (bottom). Y-axis is same as in Fig. 6F.

- Comparing to right three, left three samples show the recovery of mRNA at background level.
